# Zr_6_O_4_(OH)_4_(O_2_C-*t-*Bu)_12_ precursor uncovers how modulators govern supersaturation, nucleation, and growth of UiO-66 nanocrystals

**DOI:** 10.1039/d5sc08105j

**Published:** 2026-03-04

**Authors:** Jade M. Kemp, Jonathan S. Owen

**Affiliations:** a Department of Chemistry, Columbia University New York 10027 USA jso2115@columbia.edu

## Abstract

Pure Zr_6_O_4_(OH)_4_(O_2_C-*t*-Bu)_12_ (Zr_6_-Pivalate) is used to prepare UiO-66 nanocrystals (*d* = 19–186 nm, *σ* = 10–30%) in a hot injection synthesis. *In situ* dynamic light scattering (DLS) measurements show that nanocrystal formation is complete in 2–20 minutes, much faster than the 12–24 hours required by conventional syntheses from zirconyl chloride. The nanocrystal size monotonically increases as the benzoic acid modulator concentration and/or reaction temperature are increased and when solutes are supplied slowly. ^13^C labeled UiO-66 nanocrystals dissolve in benzoic acid modulator solution liberating terephthalic acid that is observed using nuclear magnetic resonance (NMR) spectroscopy. The solubility is size dependent (*K*_sp(d)_ = 0.7–5.7 × 10^−22^, *d* = 26–108 nm; *K*_sp(bulk)_ = 6.8 × 10^−23^), corresponding to a low surface energy (0.02 ± 0.03 to −0.001 ± 0.001 mJ m^−2^). The low surface energy explains the lack of Ostwald ripening under the synthesis conditions. The kinetics of MOF formation and the final nanocrystal size can be predicted using a homogeneous nucleation and growth mass balance model where the supersaturation is governed by a competition between the modulator and linker for the nodal cluster.

## Abstract 2

Modulator controls supersaturation, low surface energy, and slow ripening.

## Graphical abstract

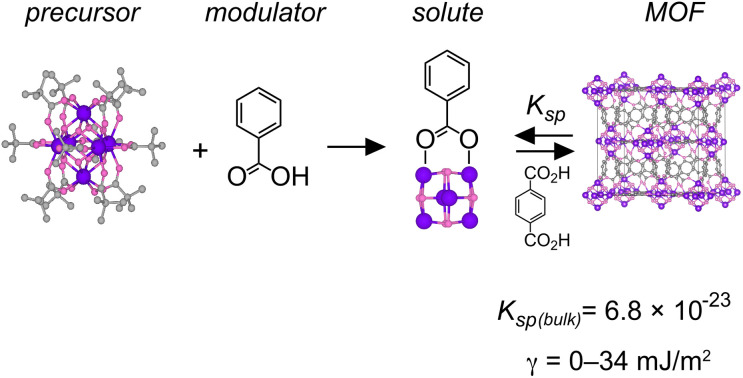

## Introduction

The high external surface area of nanometer scale metal organic frameworks (MOFs) offers advantages in gas capture,^1–3^ catalysis,^4–6^ and drug delivery.^7–9^ These applications demand control over the size, shape, and polydispersity in a high-yielding synthesis. Among nanometer scale MOFs, the UiO-66 variant can be prepared by the introduction of a carboxylic acid modulator, *e.g.* benzoic or acetic acid.^10–15^ The modulator serves multiple purposes, including (1) enhancing crystallinity by facilitating reversible Zr–O bond formation,^16,17^ (2) creating defects by its incorporation into the interior of the framework,^18,19^ (3) binding to exterior crystal facets,^15,16,20^ (4) controlling the size of MOF nanocrystals,^10,12–15,21^ and (5) reducing the importance of coordination polymer gels formed from the metal precursors^22^ or assembly of the MOF particles.^23^

Increasing modulator concentration can increase or decrease the size of MOF crystals.^15^ In some cases a non-monotonic relationship between modulator concentration and nanocrystal size is observed.^24,25^ A see-saw model has been proposed to explain these observations that is based on coupled equilibria between ligand deprotonation and metal–ligand bond formation.^24^ Alternative models have been used to describe MOF formation, including continuous nucleation,^26^ rapid nucleation,^23^ and aggregative growth,^27^ which further complicates the interpretation of the connection between modulator concentration and nanocrystal size and yield.

Modulators may also influence the mechanism and kinetics of nodal cluster (Zr_6_O_4_(OH)_4_) production, which forms *in situ* from zirconium tetrachloride or zirconyl chloride, a tetrameric hydrate.^28^ Conversion of these starting materials to clusters uses water present in the reaction medium,^23,29^ the concentration of which influences the size and reaction kinetics of the nanocrystal formation.^10,30–32^ While the water present in dimethylformamide (DMF) can provide the needed oxo units, it can also hydrolyze DMF,^33^ producing formic acid, a well-known modulator. In these ways, assembling the nodal cluster *in situ* complicates the formation of UiO-66.

Moreover, the kinetics of the nodal cluster assembly appear to be slower than the assembly of the framework. Thus, ZrOCl_2_ and ZrCl_4_ are precursors and must undergo conversion to solutes prior to MOF formation. The synthesis of nodal clusters with monotopic carboxylate ligands typically requires more than 12 hours, a timeframe that is similar to syntheses of UiO-66,^34^ and much slower than the exchange of carboxylate ligands on (Zr_6_O_4_(OH)_4_) surfaces, which has been reported to be rapid at room temperature.^35^ It is therefore difficult to disentangle the node forming kinetics from the MOF crystal growth.

Existing mechanistic models do not address the node assembly kinetics nor whether the node assembly influences the yield of the MOF. With these limitations in mind, we sought to explore the influence of modulators on the assembly of UiO-66 by first preparing the nodal cluster, a so-called secondary building unit (SBU) approach, and carrying out the synthesis under anhydrous conditions. That approach distinguishes this work from prior mechanistic studies of colloidal crystal nucleation and growth where ZrOCl_2_ and ZrCl_4_ precursor conversion limits the kinetics of solute generation.

Several Zr_6_O_8_H_4_(O_2_CR)_12_ clusters have been synthesized and structurally characterized,^36^ including Zr_6_O_4_(OH)_4_(O_2_C-*t*-Bu)_12_ (Zr_6_-Pivalate), which is readily obtained in high yield on large scales as single crystals suitable for X-ray crystallography. The high purity of the Zr_6_-Pivalate ensures the absence of other oxo-zirconium contaminants that may complicate our results. Zr_6_-Pivalate enables the synthesis of bulk UiO-66 at higher zirconium concentrations, a significant advance that we sought to translate to a high yielding synthesis of nanocrystals.^37^ Beginning with a preformed nodal cluster allows us to more directly study the influence of the modulator on the kinetics of crystal nucleation and growth, which proved to be rapid.

## Results

**Scheme 1:**
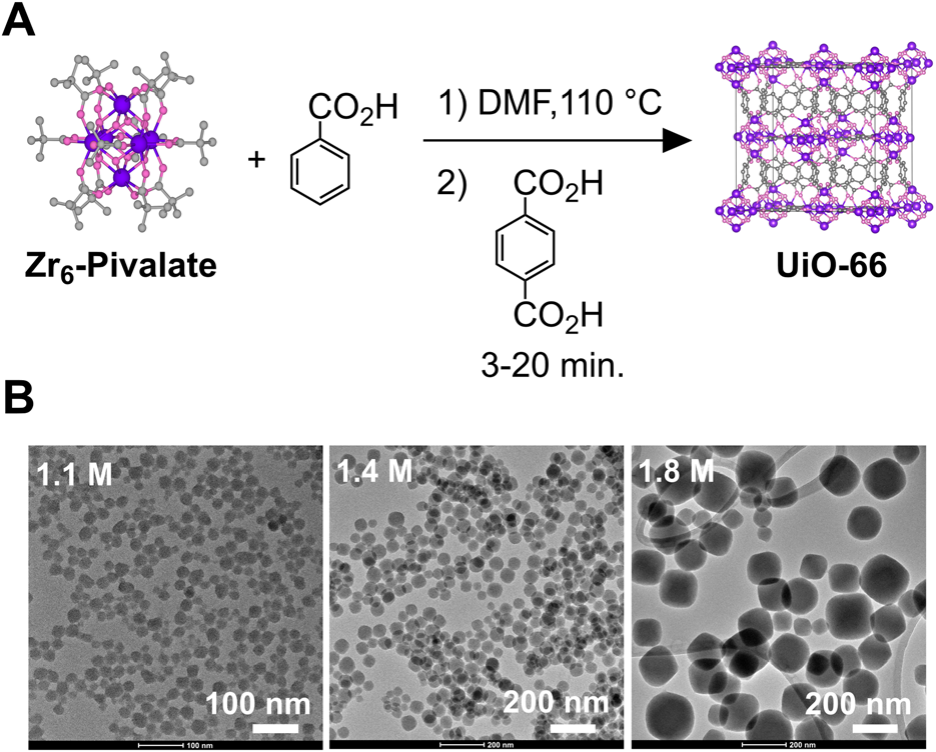
(A) Hot injection method for the synthesis of UiO-66 nanocrystals. The reaction Zr_6_-Pivalate concentration is 0.014 M (Zr), benzoic acid concentration ranges from 1.0 to 1.9 M, and terephthalic acid concentration is fixed at 0.064 M. (B) TEM images of UiO-66 nanocrystals synthesized at various modulator concentrations.

**Fig. 1:**
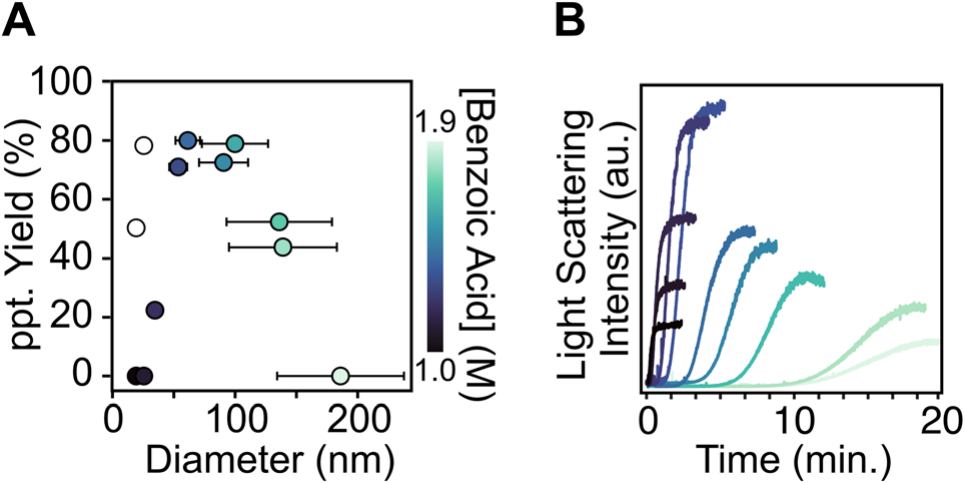
(A) Change in nanocrystal diameter *vs.* precipitate yield following centrifugation (see Section S1 and S2 for details). Error bars represent *σ* as the absolute standard deviation measured from TEM images (*n* = 200). Empty circles are yield values obtained *via* the anti-solvent precipitation method (see Section S1 for details). (B) Scattering intensity over time of UiO-66 nanoparticles synthesized *via* the hot injection method at various modulator concentrations.

Gram quantities of Zr_6_-Pivalate are prepared as described previously under conditions that optimized the formation of X-ray quality crystals (Section S1 for details).^37^ This strategy ensures the purity of our starting material, which is otherwise difficult to verify. Although insoluble in most solvents, Zr_6_-Pivalate completely dissolves in anhydrous dimethylformamide and benzoic acid (1–3 M) with heat. Under these conditions, we assume that carboxylate exchange with the benzoic acid produces a soluble benzoate capped nodal cluster (Zr_6_-Benzoate). Similar carboxylate ligand exchange reactions have been demonstrated by others without degradation of the Zr_6_O_4_(OH)_4_ core.^35,36^ “Hot injection” of terephthalic acid into this solution causes the rapid formation of colloidal UiO-66, as a translucent, colorless, suspension. Scheme 1B and Fig. 1A display nanocrystals collected following the washing procedure, and in some cases following functionalization with oleic acid and oleylamine (see Section S1 for details).

The final size steadily increases as the concentration of the benzoic acid increases, rather than the “see saw” dependence described elsewhere. The polydispersity increases to approximately 30% (*σ*/*µ*) as the modulator concentration is raised to 1.9 M (Fig. S3–S12). The size of the nanocrystals is also sensitive to temperature, increasing from an average diameter of 54 to 114 nm as the temperature is increased from 100 °C to 130 °C ([benzoic acid] = 1.4 M) (Fig. S14 and S15).

To explore the influence of solute supply kinetics on the nanocrystal size, a series of reactions were conducted by slowly adding terephthalic acid into an otherwise typical synthesis. Zr_6_-Pivalate is dissolved in a solution of benzoic acid (1.4 M) in anhydrous DMF to which a solution of terephthalic acid is slowly added *via* syringe pump over a period of 5, 10, 50, or 100 minutes (Table S4). The nanoparticle size increases as the injection rate slows or the reaction temperature increases (Fig. S16–S19). The polydispersity increases with higher temperatures and slower addition rates, independent of the modulator concentration (Fig. S15).

Dynamic light scattering (DLS) was used to track the formation of MOFs following the hot injection of terephthalic acid (Fig. 1B). An induction period or nucleation delay is observed after injection, followed by a steep increase, and finally a plateau in the scattering intensity. The delay increases from a few seconds to 10 minutes at the highest benzoic acid concentration (Fig. S2). Previous work found a similar relationship between the modulator concentration and the induction delay time.^31^ A “reaction endpoint” can be defined by the point at which the scattering intensity plateaus. The plateau suggests the yield of UiO-66 has reached 100% and the colloidal dispersion is stable against growth by ripening at this point.

The evolution of the final scattering intensity tracks with the amount of precipitate that can be isolated by centrifugation (Fig. 1). From [benzoic acid] = 1.0 to 1.4 M, the final scattering intensity increases, as does the size of the nanocrystals and the ease with which they can be removed by centrifugation without adding an anti-solvent. Beyond [benzoic acid] = 1.5 M, the final scattering intensity decreases despite the larger sizes produced and the *r*^6^ dependence of the Rayleigh scattering probability. At this benzoic acid concentration, the framework is partially soluble and does not form completely.

An important distinction is made here between the framework *solubility,* which refers to the disintegration of a solid framework into molecularly dissolved solutes (*e.g.* benzoate ligated zirconium clusters and terephthalic acid)*,* and the particle *dispersibility* (*e.g.* nanocrystals suspended in liquid). Note that this terminology is distinct from recent literature which mixes colloidal and molecular taxonomies and uses the terms “particle solubility” or “colloidal solubility” to describe colloidal dispersions.^38,39^ Here, we reserve the term solubility to address the equilibrium between molecular solutes and the framework solid.

At low benzoic acid concentrations, and correspondingly small crystal sizes, the precipitate yield is limited by the high dispersibility of the nanocrystals, which remain suspended in the DMF solution (filled circles, Fig. 1A) unless a lower dielectric antisolvent (*e.g.*, tetrahydrofuran) is added (see Section S1 for details). The yield of the small nanocrystals isolated by centrifugation can be increased to over 80% by inducing nanocrystal aggregation *via* an antisolvent (empty circles, Fig. 1A).

**Fig. 2:**
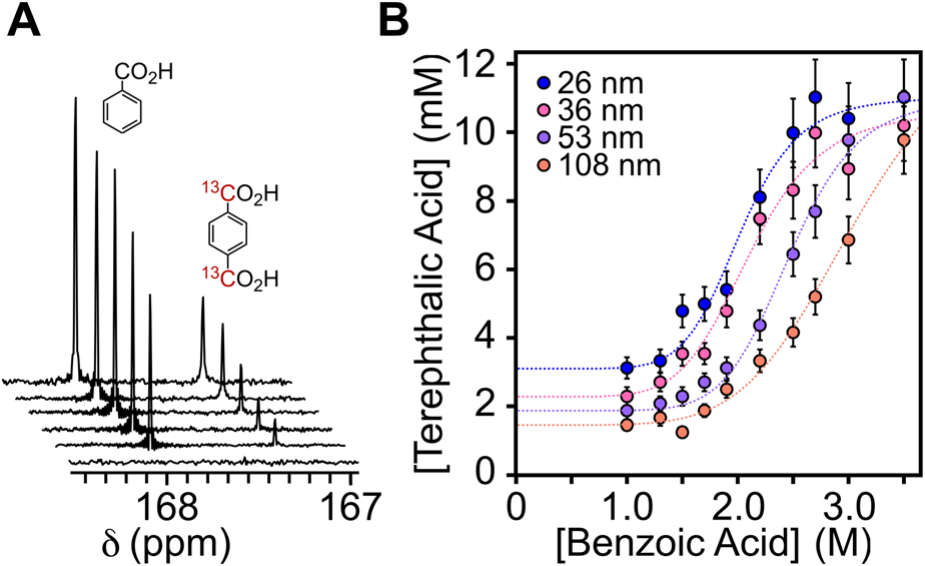
(A) ^13^C NMR of the dissolution of UiO-66 nanocrystals at increasing [benzoic acid]. (B) Terephthalic acid concentration determination *via* the dissolution of nanocrystals at multiple sizes in increasing amounts of benzoic acid. Error values determined from NMR peak integration are assumed to be 10% or the inverse of the signal-to-noise ratio (SNR) if SNR >10%.

To investigate the solubility of UiO-66 in benzoic acid solution, four sizes of ^13^C-labeled UiO-66 nanocrystals (*d* = 26 nm, 36 nm, 53 nm, and 108 nm) were dissolved in benzoic acid and DMF and the solubility determined using ^13^C NMR spectroscopy (Fig. 2A). The NMR signals corresponding to the MOF nanocrystal are not visible, presumably because of the slow tumbling and heterogeneous broadening typical of nanocrystals. However, the sharp signal of free terephthalic acid is compared with an internal standard to measure the quantity of ^13^C-labeled terephthalic acid liberated from the MOF at each benzoic acid concentration. The signal of free terephthalic acid increases as [benzoic acid] increases from 0 to 1 M, followed by a plateau at 10–30% of the total expected terephthalic acid concentration. We assign this first plateau to the release of terephthalic acid ligands coordinated to the nanoparticle surface (see Section S4 for details). Smaller nanocrystals liberate greater amounts of terephthalic acid, which is consistent with their larger external surface to volume ratio.

As [benzoic acid] increases, the solution becomes less cloudy, indicating that an increasing amount of MOF is dissolved. By [benzoic acid] = 6M, the UiO-66 framework is completely disintegrated, liberating all the linker units within the nanocrystals. This results in a clear, homogeneous solution. The reversibility of the dissolution equilibrium was tested by adding terephthalic acid to a solution of partially dissolved MOF. The added terephthalic acid induces crystallization of fresh MOF, indicating that the dissolution is reversible and that the nodal clusters remain intact (see Section S4 for details).

**Fig. 3:**
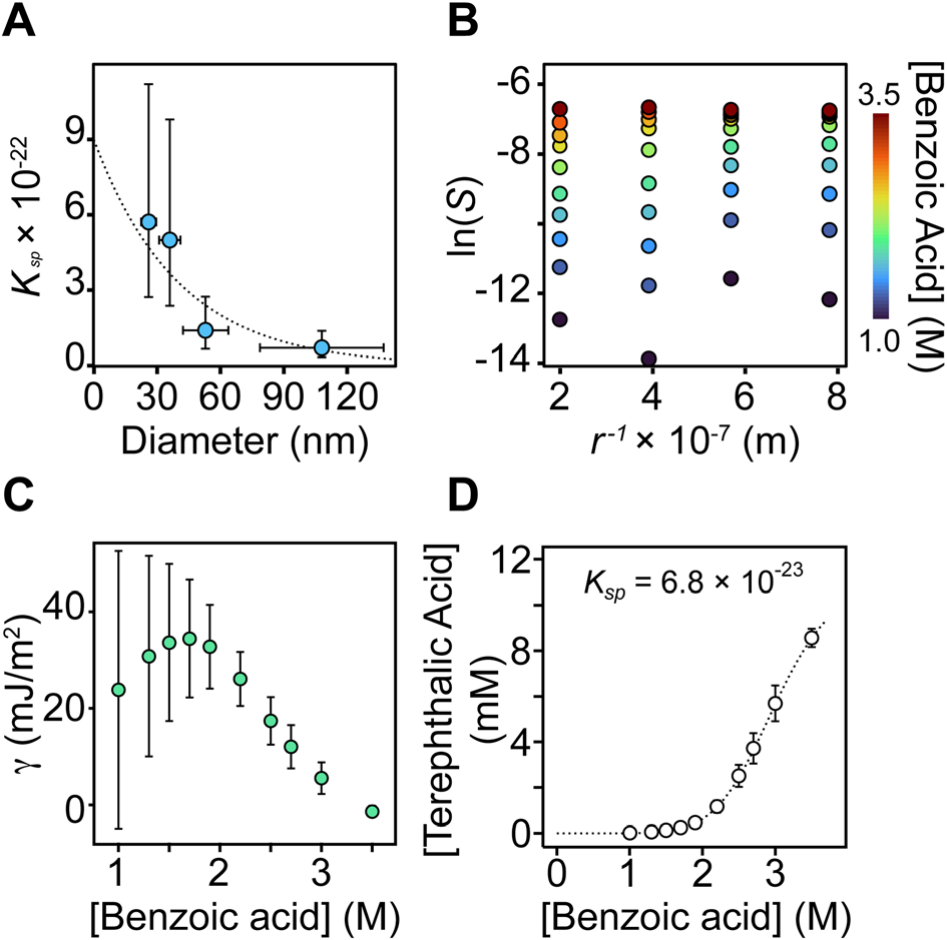
(A) Calculated *K*_sp_ at each nanoparticle diameter size. Horizontal error bars represent *σ* as the absolute standard deviation measured from TEM images (*n* = 100). Vertical error bars determined from NMR peak integration assumed to be 10%. The *K*_sp_ is determined from the subtraction of surface terephthalate ligands (see SI Section S4 for details). (B) The natural log of the solubility *vs. r*^−1^ at each nanocrystal size and [benzoic acid] (M). (C) The surface energy at each [benzoic acid] (M). Error bars incorporate the standard error of the slope of (B). (D) Bulk *K*_sp_ determined from the *y*-intercept of (B) across all benzoic acid concentrations. Error bars incorporate the standard error of the *y*-intercept. See SI Section S4 for details.

The titration curves reveal a size-dependent framework solubility from which a *K*_sp_ for each size and for bulk UiO-66 can be determined (see Discussion Section). Interestingly, there was no evidence of Ostwald ripening under these conditions nor after prolonged heating at the synthesis temperature, despite the high concentration of MOF subunits in the solution (Fig. 3C, S23, and S24).

## Discussion

### Solubility

The size dependent solubility observed in Fig. 2B can be attributed to the large surface area to volume ratio change over the size range studied (26–108 nm). While modulator induced defects may influence the solubility^40^ and can be size dependent,^41^ a narrow range of [benzoic acid] was used to prepare the sizes in Fig. 2. Defects associated with hydrolysis should also be minimal under the anhydrous conditions used here. Hence, we attribute the size dependent solubility to the 4-fold decrease in the surface area to volume ratio across the range of sizes studied. Nonetheless, the measured solubilities are artificially inflated by defects compared to the bulk MOF, a subject that is beyond the scope of the current study.

In light of these complications, we interpret the size dependent solubility using the Ostwald–Freundlich equation (eqn (1), see SI S4 for details).^42,43^ The surface energy (*γ*) can be calculated from the solubility (*S* = solubility, molkg^−1^) at each [benzoic acid] where *V*_m_ is the molar volume, *R* is the universal gas constant, *r* is radius, and *T* is temperature.1
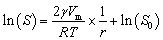


Plotting the ln(*S*) *versus* 1/*r* at each [benzoic acid] reveals the size dependence of the solubility – smaller sizes have greater solubility (Fig. 3B). The slope of the linear fit to these data points provides the surface energy, while the intercept provides the bulk solubility of UiO-66 at each [benzoic acid]. These measures of the bulk MOF solubility provide a *K*_sp_ = 6.8 × 10^−23^ (lower bound 5.7 × 10^−23^, upper bound 9.6 × 10^−23^), which was extracted from the fit of solubility *vs.* [benzoic acid] shown in Fig. 3D.

As the [benzoic acid] increases, the surface energy decreases to near zero (Fig. 3C). This indicates that benzoic acid adsorbs to the surface exothermically and explains the increased dispersibility of nanocrystals in benzoic acid solution. X-ray structures of nodal clusters with carboxylic acids hydrogen-bonded to the µ_3_-oxo units support that picture.^35^ All of these point to an exothermic adsorption of benzoic acid to the nanocrystal surface.

In surfactant stabilized dispersions such as oil/water emulsions, surfactant adsorption can reduce *γ* below 10 mJ m^−2^ and produce indefinitely stable dispersions with low rates of Ostwald ripening.^44^ The surface energies measured here are similarly low and explain the slow rate of Ostwald ripening, which is proportional to the surface tension in Lifshitz–Slyozov–Wagner theory (Fig. S23). The low *γ* also suggests that nucleation kinetics are weakly affected by the size dependence of the solubility, which is the thermodynamic basis for a critical nucleus in classical nucleation theory. This distinguishes MOFs from other crystals where a larger surface energy leads to a short burst of nucleation.

A low surface energy is perhaps unsurprising given that the surface-ligand and node-linker bonding interactions are both Zr-carboxylate interactions. In addition, the large unit cell of MOFs reduces interactions between surface ligands, which strongly influences the energetics of ligand binding in crystals with small unit cells, such as silicon,^45^ cadmium selenide,^46^ or gold.^47^ Thus, it can be expected that other MOFs will have similarly small surface energies, provided the surface ligands are similar to the linker. Modifying the choice of modulator might be used to influence the surface energy by strengthening or weakening the bonding interaction with the MOF surface relative to terephthalic acid. This may directly impact the duration of nucleation, ripening, and size distribution focusing, all of which govern the polydispersity. Alternative modulators that increase the nanoparticle surface energy could shorten the nucleation period and create narrower size distributions.

### Size control

Beginning from Zr_6_-Pivalate greatly reduces the reaction time needed to synthesize UiO-66 nanocrystals (2–20 minutes) compared to conventional methods that heat ZrOCl_2_ or ZrCl_4_ with terephthalic acid for more than 12 hours.^36,48^ Similarly long reaction times are used to prepare Zr_6_-Pivalate or other Zr_6_ clusters.^34^ Hence formation of the nodal cluster is an important rate limiting step in conventional syntheses of UiO-66 and likely other MOFs derived from Zr_6_O_8_ nodes. Here, kinetics are much faster and limited by the assembly of preformed nodal clusters. This difference may explain why we do not observe the “see saw” dependence of the nanoparticle size on modulator concentration reported previously.^24,25^

**Scheme 2:**
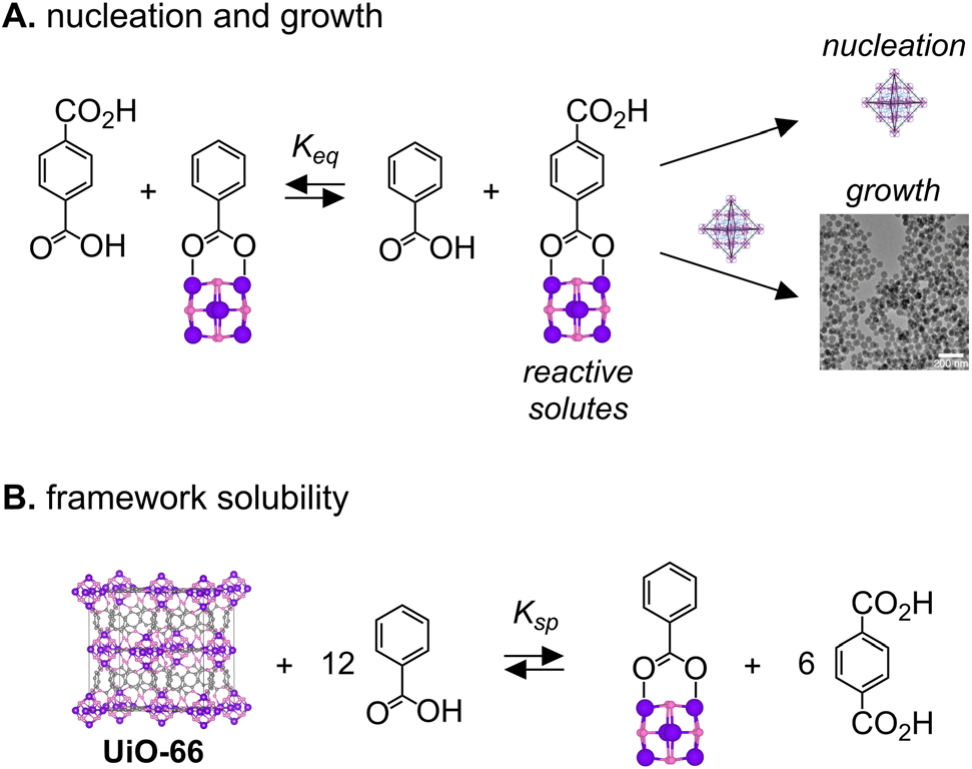
(A) The equilibrium of the modulator-capped and linker-capped hexa-zirconium cluster has a direct effect on the nucleation and growth kinetics of nano-MOF formation. (B) Solubility equilibrium of modulator-capped and linker-capped hexa-zirconium cluster during nano-MOF reaction.

The influence of the modulator concentration on the nanocrystal size can be understood by considering the supersaturation during nucleation and growth. Scheme 2A illustrates how benzoic acid changes the concentration of terephthalate coordinated nodes that precede the MOF nucleation and growth steps. The concentration of the terephthalate ligated cluster will influence the kinetics of nucleation and growth. The modulator type and concentration determines the position of that equilibrium. Carboxylate exchange is rapid at room temperature^35^ and therefore unlikely to limit the precipitation kinetics observed here. A similar exchange explains the solubility of the MOF in benzoic acid solution as shown in Scheme 2B.

These equilibria can explain three features of the *in situ* DLS curves: (1) the time before the onset of light scattering, “the induction delay time” (Fig. S2), (2) the slope of the light scattering intensity curve following the induction delay, and (3) the intensity of the light scattering at the reaction end point. In syntheses conducted above [benzoic acid] = 1.6 M, a significant quantity of the UiO-66 lattice dissociates at equilibrium, decreasing the yield and thereby decreasing the light scattering intensity at the reaction endpoint (Fig. 1). Increasing benzoic acid also lowers the supersaturation, which slows the nucleation and the growth processes and explains the long induction delay times and decreasing precipitation rate shown in Fig. 1B. Hence, measuring the MOF solubility in the modulator solution can help optimize the final size, reaction time, and yield.

Scheme 2A also illustrates how reactive solutes are consumed by two competing crystallization manifolds: nucleation and growth. The ratio of these two paths dictates the extent of nucleation and, given that ripening is slow under these conditions, the final nanocrystal size at a given yield.^49–55^ Nucleation processes can be expected to have a stronger sensitivity to the supersaturation than the growth kinetics. Therefore, reducing the supersaturation will reduce the number of nuclei and produce particles of a larger final size. This relationship explains the sizes obtained at different [benzoic acid] in Scheme 1 and Fig. 1A.

Rapid carboxylate exchange at the hexa-zirconium node, the absence of light scattering prior during the induction delay, and the relatively high solubility of the MOF under synthesis conditions all indicate a homogeneous nucleation and growth mechanism is operative here. Size control in homogeneous nucleation and growth reactions can be analysed using so-called nucleation mass balance models (Eqn. (2)). These models illustrate how the rate of solute generation during nucleation (*Q*_0_, mol L^−1^ s^−1^) and the rate of solute consumption by crystal growth (*v*_*n*_, nm^3^ L^−1^ s^−1^) control the number of crystals produced by nucleation (*n*_f_, L^−1^).^56^2
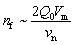


While the present hot injection synthesis establishes maximum supersaturation following injection, other colloidal synthesis methods control the rate of solute generation by slow addition of reagents with a syringe pump or by utilizing synthesis precursors that undergo conversion to solutes *in situ*. In some cases the conversion reactivity can be tuned by adjusting the precursor substitution pattern.^49–55^ These methods have shown that the rate of solute supply influences the extent of nucleation as described by eqn (2).^56–58^

**Fig. 4:**
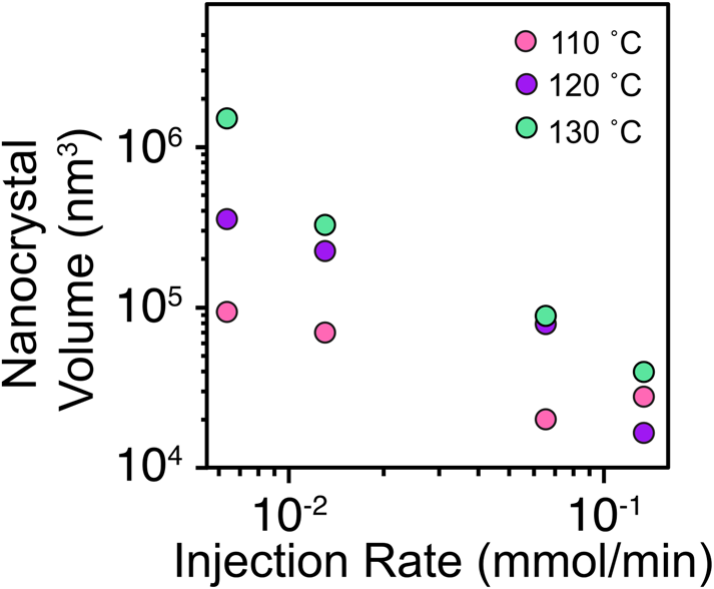
Effect of the injection rate of the linker and temperature on the nanocrystal volume. The average nanocrystal volume was calculated *via* the octahedron volume formula.

Slow injection of terephthalic acid into the solution of nodal clusters at a single modulator concentration ([benzoic acid] = 1.4 M) has a similar influence on the present synthesis. The average nanocrystal volume is plotted *versus* the injection rate of the linker at several reaction temperatures in Fig. 4. Slow injection lengthened the total reaction time to as much as 105 minutes in some cases, a time frame that is much slower than crystal growth in the hot injection reactions studied by DLS. The steady decrease in nanocrystal volume with increasing addition rate is qualitatively consistent with the homogeneous nucleation and growth mechanism shown in Scheme 2A and nucleation mass balance models.

The increase of size with temperature is also similar to previous studies on colloidal metal chalcogenides,^49–52^ indium phosphide^55^ nanocrystals, and the crystallization of proteins.^59^ Although the precise origin of the temperature effect is uncertain, and a variety of temperature dependences on crystal nucleation have been reported, higher temperatures under our conditions disfavor nucleation and favor crystal growth. Adjusting the temperature provides a valuable method to tune size without modifying the modulator concentration, a strategy that could improve control over defect densities.

## Conclusion

A preformed Zr_6_-Pivalate cluster and anhydrous DMF avoid the slow kinetics of forming nodal clusters during the assembly of UiO-66 nanocrystals and its influence on defects caused by hydrolysis of the MOF or the formation of formic acid from the DMF. The resulting conditions provide reproducible and tunable reaction kinetics that occur within minutes following hot injection of the terephthalic acid linker. The connection between the modulator concentration and the nanocrystal size are the result of changes to the supersaturation following hot injection. Moreover, we find that the UiO-66 solubility is weakly size dependent, corresponding to a low surface energy.

The lack of detectable scattering prior to the induction period in the DLS measurements, the relatively high solubility of the framework during synthesis, and the correlation between solute supply and average nanoparticle size all support a homogeneous nucleation and growth mechanism. The predictability of that mechanism, and the high yields obtained, speaks to the advantages of using preformed nodal clusters in MOF synthesis and the value of measuring MOF solubility in the modulator solution. However, the low surface energies measured here suggest that the kinetics of nucleation will be slow and lead to broad size distributions. This observation is consistent with the lack of Ostwald ripening during growth. Both characteristics differentiate MOF crystallization from conventional colloidal crystal growth mechanisms. The relatively weak dependence of the thermodynamics on the size makes the mechanism here more akin to a polymerization than a classical nucleation and growth process. Such behavior may be common to other MOFs with similarly large unit cells and grown with modulators that are structurally similar to the linkers.

## Author contributions

The manuscript was written through contributions of all authors.

## Conflicts of interest

There are no conflicts to declare.

## Supplementary Material

SC-OLF-D5SC08105J-s001

## Data Availability

All data is available in the supporting information (SI) that accompanies this manuscript. Supplementary information is available. See DOI: https://doi.org/10.1039/d5sc08105j.
