# Supplementary material for: Zr6O4(OH)4(O2C-t-Bu)12 precursor uncovers how modulators govern supersaturation, nucleation, and growth of UiO-66 nanocrystals
Source: Chem Sci. 2026 Mar 4;17(17):8719–25. doi: 10.1039/d5sc08105j (PMC12980884; doi:10.1039/d5sc08105j)
Supplement: SC-017-D5SC08105J-s001 [file SC-017-D5SC08105J-s001.pdf]

Supplementary Information (SI) for Chemical Science.
This journal is © The Royal Society of Chemistry 2026
Evaluation Only. Created with Aspose.Pdf. Copyright 2002-2014 Aspose Pty Ltd.

Supplementary Information for

$Zr_6O_4(OH)_4(O_2C\text{-}t\text{Bu})_{12}$ precursor uncovers how modulators govern supersaturation, nucleation, and growth of UiO-66 nanocrystals

Jade M. Kemp,[1] Jonathan S. Owen[1]*

[1]Department of Chemistry, Columbia University, New York, NY 10027, United States

*To whom correspondence may be addressed: jso2115@columbia.edu

Section S1: Experimental Section

Section S2: Nanocrystal Characterization

Section S3: Yield Determination

Section S4: Nanocrystal Solubility Measurements

Section S5: Modeling

Evaluation Only. Created with Aspose.Pdf. Copyright 2002-2014 Aspose Pty Ltd.

S1. EXPERIMENTAL SECTION

1. Materials and Methods

1.1. General Considerations

All operations were completed in air unless otherwise specified. Pivalic acid (99%), benzoic acid (ACS reagent, ≥ 99.5%), tetrahydrofuran (ACS reagent, ≥ 99.0%), oleic acid (≥ 99%), toluene (ACS reagent, ≥ 99.5%), terephthalic acid-2,2'-$^{13}C_2$ (99 atom % ^{13}C), 2-propanol (anhydrous, 99.5%), and methyl acetate (ReagentPlus®, 99%), were purchased from Sigma-Aldrich and used a received. Zirconium (IV) isopropoxide isopropanol complex (99.9% trace metals basis) were purchased from Sigma-Aldrich and stored in a nitrogen-filled glovebox. *N,N*-Dimethylformamide (ACS reagent, ≥ 99.8%) was purchased from Sigma-Aldrich and stored over molecular sieves. *N,N*-Dimethylformamide (anhydrous, 99.8%) was purchased from Sigma-Aldrich and stored in a Strauss flask under argon. Oleylamine (technical grade, 70%) was purchased from Sigma-Aldrich and purified via fractional distillation according to literature procedure.[1] Toluene-d_8 (99+ atom % D) was purchased from Thermo Scientific. Hexanes (certified ACS) was purchased from Thermo Scientific, dried over CaH_2, distilled, and stored in a nitrogen-filled glovebox over molecular sieves. Terephthalic acid (99+%) and methyl sulfone (98%) were purchased from Acros Organics. Zirconium (IV) chloride (99.95%+ Zr) was purchased from Strem and stored in a nitrogen-filled glove box. Zirconium (IV) n-propoxide (70% w/w in n-propanol) was purchased from Alfa Aesar. Dimethyl sulfoxide-d_6 (D, 99.9%) was purchased from Cambridge Isotope Laboratories.

Evaluation Only. Created with Aspose.Pdf. Copyright 2002-2014 Aspose Pty Ltd.

1.2. Instrumentation

Single-Crystal X-ray Diffraction (SCXRD) patterns were measured using an Agilent SuperNova single-crystal X-ray diffractometer.

Transmission electron microscopy (TEM) images were collected on a FEI Talos F200X. Samples were prepared by drop casting a diluted MOF suspension in toluene on a Lacey carbon grid (Ted Pella). Grids were dried at room temperature before introducing them into the microscope.

Dynamic Light Scattering (DLS) was measured using a Cordouan Technologies VASCO KIN.

Thermogravimetric Analysis (TGA) was conducted using a TA Instruments Q500 Thermogravimetric Analysis.

Nuclear Magnetic Resonance (NMR) spectra was collected using a Bruker 500 MHz spectrometer. All ^{13}C spectra were referenced to deuterated toluene solvent signals (137.83 ppm).

Powder X-Ray Diffraction (PXRD) patterns were measured using a PANalytical X'Pert powder X-ray diffractometer.

Evaluation Only. Created with Aspose.Pdf. Copyright 2002-2014 Aspose Pty Ltd.

2. Experimental description

2.1 Oxo-zirconium cluster

2.1.1 $Zr_6O_4(OH)_4(OOCC(CH_3)_3)_{12}$ Cluster. Procedure was adapted from synthetic method published by Jerozal et al.[2] To a 2 L round bottom flask (RBF), $ZrCl_4$ (5.8340 g, 25.0 mmol) and 1.00 L of DMF were added. Mixture sonicated to dissolve $ZrCl_4$. Pivalic acid (127.75 g, 1.25 mol) and water (1.35 mL, mmol) were added. RBF equipped with a reflux condenser and argon-filled balloon. The reaction mixture was placed in a mantle and heated to 120 °C using a Digi-Sense Temperature Controller. After 48 hours, the reaction was cooled to room temperature and crystalline precipitate was isolated with frit. Precipitate washed with DMF, THF, and dried under vacuum overnight at 100 °C to yield white crystals (5.18 g, 65% yield). Structure confirmed via SCXRD with published unit cell parameters.[2]

2.2 MOF nanocrystal synthesis

2.2.1 Hot Injection UiO-66 Nanocrystals (1.0 M and 1.1 M benzoic acid)

To a 20 mL vial, $Zr_6O_4(OH)_4(OOCC(CH_3)_3)_{12}$ (0.0442 g, 0.0233 mmol) and benzoic acid (1.2212g, 0.0100 mol) were added with 8 mL of anhydrous DMF and a small stir bar. The vial was capped with a septum and an argon-filled balloon. Reaction stirred at 300 rpm for 40 minutes at 110 °C on a metal heating plate. In a separate 20 mL vial, terephthalic acid (0.1070 g, 0.644 mmol) and 2 mL of anhydrous DMF were added and heated to 110 °C on a metal heating plate. The terephthalic acid solution was injected and reaction progress was monitored via continuous, *in situ* dynamic light scattering (DLS). Because the nanocrystals do not precipitate from solution readily, an anti-solvent precipitation method to isolate the nanocrystals was established. The reaction mixture was divided evenly into two centrifuge tubes, followed by 25 mL of THF to precipitate out the nanocrystals. Precipitate is isolated via centrifugation at 11000 rpm for 20 minutes. Precipitate is washed once with DMF and THF. Nanocrystals with a diameter of 19.3 nm were isolated as a white powder. Procedure is repeated with benzoic acid (1.3434 g, 0.011 mol) to produce nanocrystals with a diameter of 25.5 nm.

2.2.2 Hot Injection UiO-66 Nanocrystals (1.2 – 1.9 M benzoic acid)

To a 20 mL vial, Zr6O4(OH)4(OOCC(CH3)3)12 (0.0442 g, 0.0233 mmol) and benzoic acid (1.4654 g, 0.012 mol) were added to 8 mL of anhydrous DMF and small stir bar. The vial was capped with a septum and argon filled balloon. Reaction stirred at 300 rpm for 40 minutes at 110 °C on a metal heating plate. In a separate 20 mL vial, terephthalic acid (0.1070 g, 0.644 mmol) and 2 mL of anhydrous DMF were added and heated to 110 °C on a metal heating plate. The terephthalic acid solution was injected and reaction progress was monitored via, continuous *in situ* DLS. Reaction is stopped when the scattering intensity plateaus for 90 seconds. Precipitate is isolated via centrifugation at 11,000 rpm for 20 minutes. Precipitate is washed once with DMF and THF. Nanocrystals with a diameter of 34.6 nm were isolated as a white powder. To increase the size of the nanocrystals, the benzoic acid concentration or temperature was increased, while keeping all other variables constant.

Evaluation Only. Created with Aspose.Pdf. Copyright 2002-2014 Aspose Pty Ltd.

Table S1. Alternative hot injection reaction conditions

[Benzoic Acid] (M)	Benzoic Acid mass (g)	Temperature (°C)	Average Nanocrystal Diameter (nm)
1.3	1.5876	110	53.6
1.4	1.7097	110	61.4
1.5	1.8318	110	90.6
1.6	1.9539	110	99.9
1.7	2.0760	110	136
1.8	2.1982	110	139
1.9	2.3203	110	186
1.4	1.7097	100	53.4
1.4	1.7097	120	77.3
1.4	1.7097	130	114

2.2.3 Slow Injection UiO-66 Nanocrystals

To a 20 mL vial, $Zr_6O_4(OH)_4(OOCC(CH_3)_3)_{12}$ (0.0442 g, 0.0233 mmol) and benzoic acid (1.7097, 0.0140 mol) were added with 8 mL of anhydrous DMF and a small stir bar. The vial was capped with a septum and an argon-filled balloon. Reaction stirred at 300 rpm for 40 minutes at 110°C on metal heating plate. In a separate 20 mL vial, terephthalic acid (0.1070 g, 0.644 mmol) and 2 mL of anhydrous DMF were added and heated to 110°C on a metal heating plate. The terephthalic acid solution was injected at a rate of 0.13 mmol/min. After injection, the reaction was heated an additional 5 minutes 15 seconds, corresponding to the time a hot injection reaction at this temperature and benzoic acid concentration would occur (acquired via DLS). Precipitate is isolated via centrifugation at 11,000 rpm for 20 minutes. Precipitate is washed once with DMF and THF. Nanocrystals with an edge length of 38.9 nm were isolated as a white powder. To increase the size of the nanocrystals, the injection rate was slowed or the temperature was increased, while keeping all other variables constant.

Table S2. Alternative slow injection reaction conditions

Injection Rate (mmol/min)	Temperature (°C)	Additional Reaction Time After Injection	Average Nanocrystal Edge Length (nm)
0.0064	110	5 min 15 sec	58.4
0.013	110	5 min 15 sec	52.7
0.064	110	5 min 15 sec	34.9
0.0064	120	4 min 50 sec	90.8
0.013	120	4 min 50 sec	78.0
0.064	120	4 min 50 sec	55.1
0.13	120	4 min 50 sec	32.7
0.0064	130	4 min 10 sec	147
0.013	130	4 min 10 sec	86.0
0.064	130	4 min 10 sec	53.7
0.13	130	4 min 10 sec	43.8

Evaluation Only. Created with Aspose.Pdf. Copyright 2002-2014 Aspose Pty Ltd.

2.4 Surface Functionalization

2.4.1. Surface functionalization method with oleic acid and oleyl amine

UiO-66 nanocrystals synthesized via the hot injection of terephthalic acid at 1.0 M and 1.1 M benzoic acid were functionalized with oleic acid and oleyl amine to decrease aggregation prior to acquisition of TEM images. Nanocrystals isolated from the anti-solvent precipitation method described above were added to a centrifuge tube with toluene (3 mL) and oleic acid (0.122 mL, 0.432 mmol) and the mixture vortexed for 1 minute. Next, oleyl amine (0.130 mL, 0.486 mmol) was added and the mixture was vortexed for 3 minutes to produce a clear dispersion. The nanocrystals were isolated via antisolvent precipitation using 15 mL of methyl acetate. Precipitate is isolated via centrifugation @11,000 rpm for 20 minutes. The precipitate is redissolved in toluene (3 mL) and methyl acetate (15 mL) is added to precipitate the nanocrystals and the suspension centrifuged at 11,000 rpm for 20 minutes. The precipitate is dispersed in toluene (5 mL), centrifuged at 8,000 rpm for 8 minutes, and the clear supernatant is collected. This method is based on previous literature.[3]

Evaluation Only. Created with Aspose.Pdf. Copyright 2002-2014 Aspose Pty Ltd.

SECTION S2. NANOCRYSTAL CHARACTERIZATION

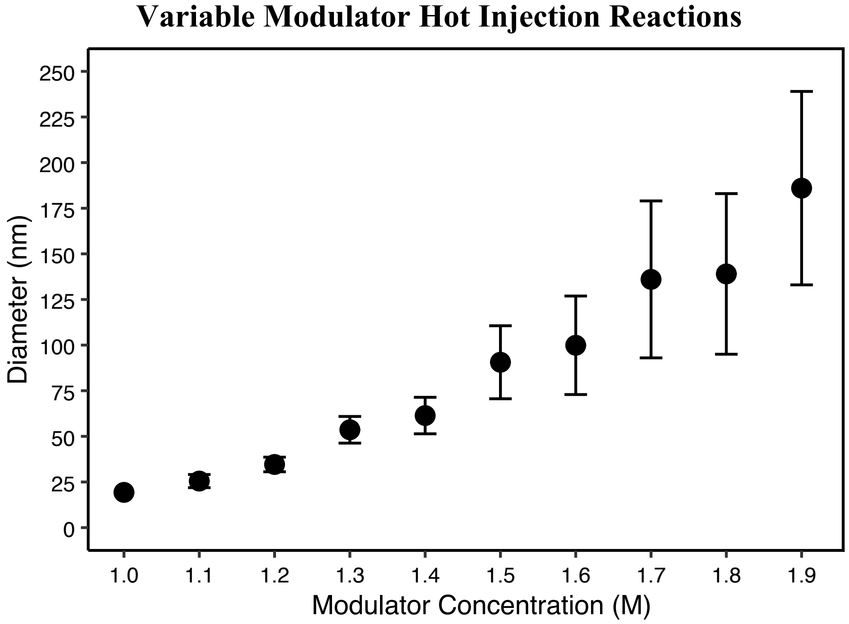

Figure S1. Change in nanocrystal diameter synthesized via the hot injection method with respect to modulator concentration. Error bars represent $\mu \pm \sigma$, where μ is the mean diameter and σ is the standard deviation.

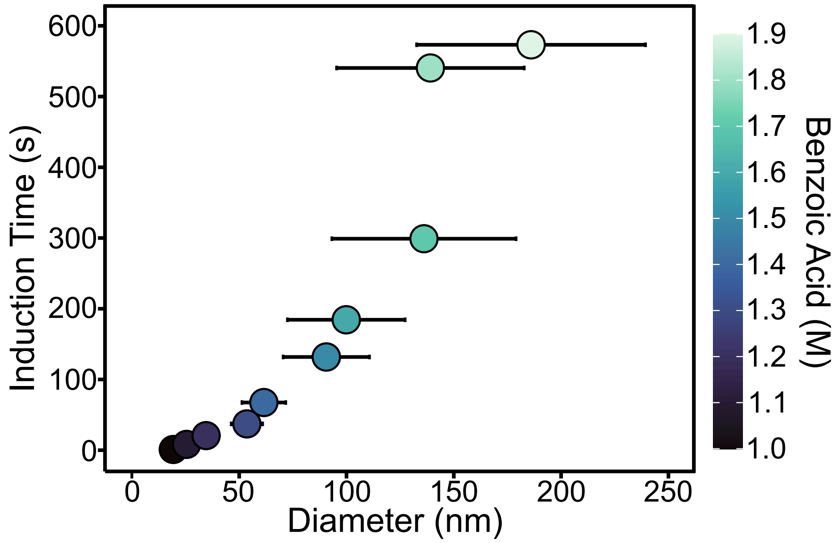

Figure S2. Induction time versus average diameter for nano-UiO-66 MOFs prepared via the hot injection of linker. Error bars represent σ as the absolute standard deviation measured from TEM images (n=200).

Evaluation Only. Created with Aspose.Pdf. Copyright 2002-2014 Aspose Pty Ltd.

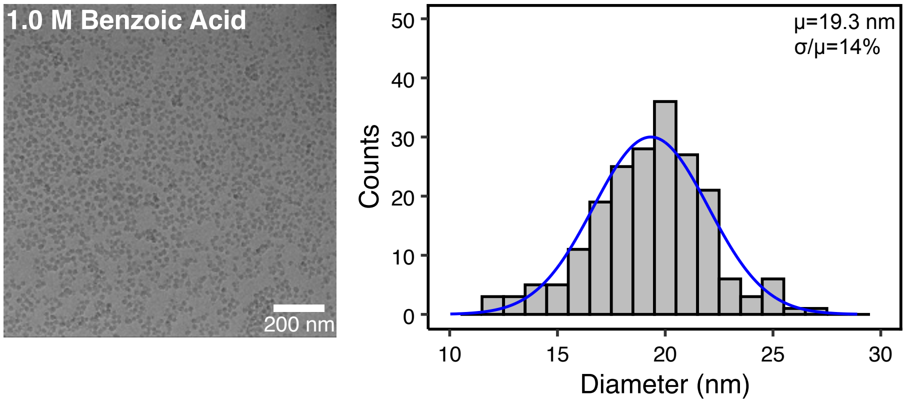

Figure S3. TEM and histogram distribution of UiO-66 nanocrystals synthesized with 1.0 M benzoic acid concentration via hot injection method at 110°C (n = 200 particles).

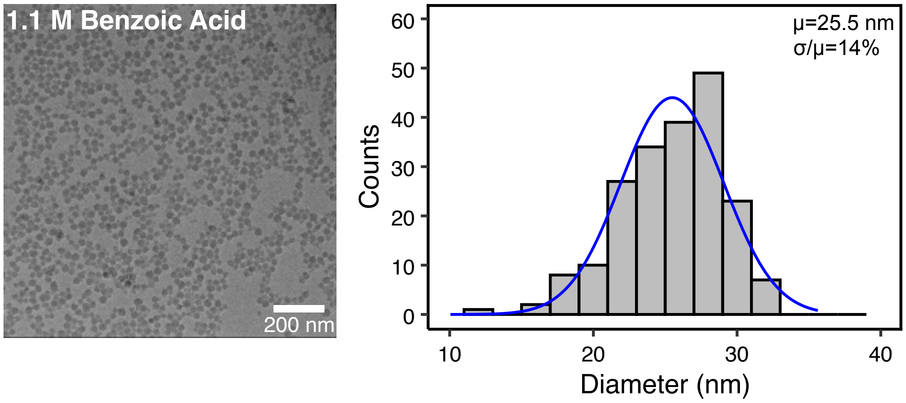

Figure S4. TEM and histogram distribution of UiO-66 nanocrystals synthesized with 1.1 M benzoic acid concentration via hot injection method at 110°C (n = 200 particles).

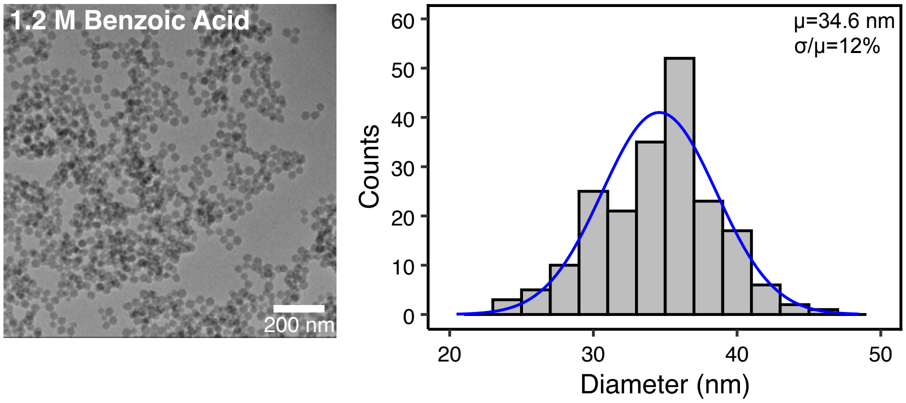

Figure S5. TEM and histogram distribution of UiO-66 nanocrystals synthesized with 1.2 M benzoic acid concentration via hot injection method at 110°C (n = 200 particles).

Evaluation Only. Created with Aspose.Pdf. Copyright 2002-2014 Aspose Pty Ltd.

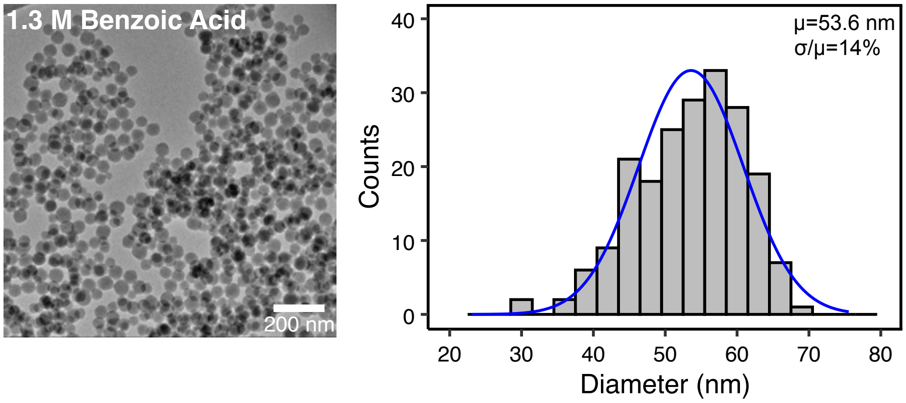

Figure S6. TEM and histogram distribution of UiO-66 nanocrystals synthesized with 1.3 M benzoic acid concentration via hot injection method at 110ºC (n = 200 particles).

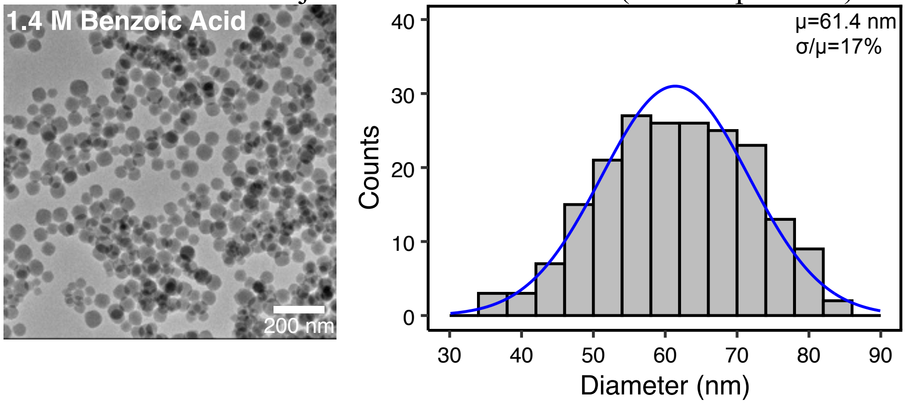

Figure S7. TEM and histogram distribution of UiO-66 nanocrystals synthesized with 1.4 M benzoic acid concentration via hot injection method at 110ºC (n = 200 particles).

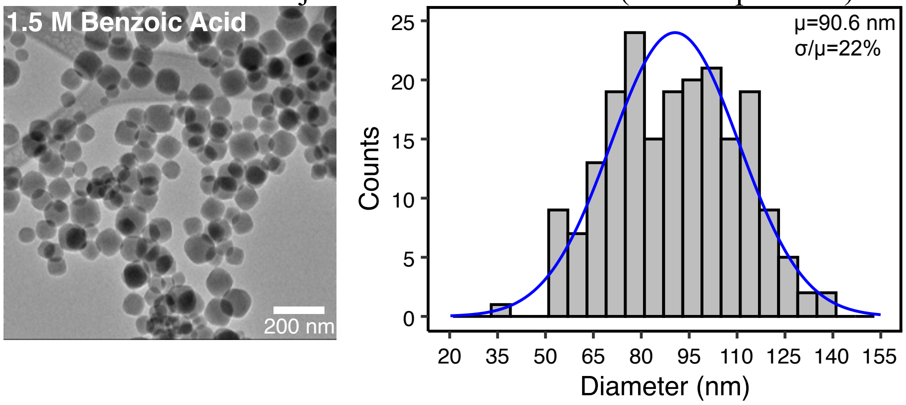

Figure S8. TEM and histogram distribution of UiO-66 nanocrystals synthesized with 1.5 M benzoic acid concentration via hot injection method at 110ºC (n = 200 particles).

Evaluation Only. Created with Aspose.Pdf. Copyright 2002-2014 Aspose Pty Ltd.

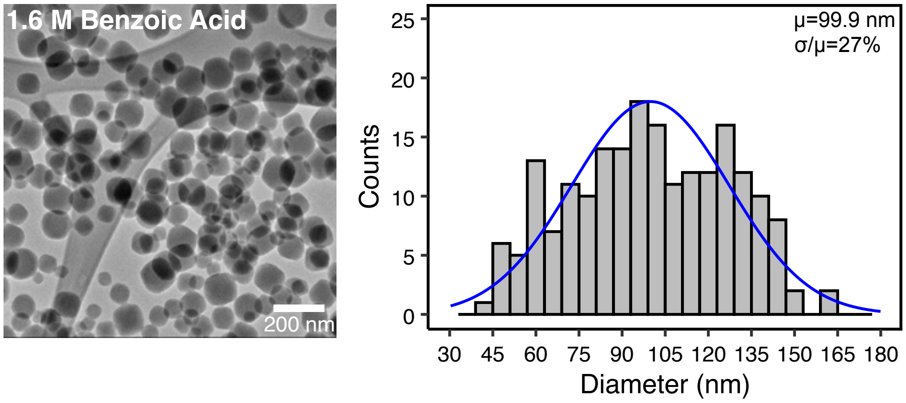

Figure S9. TEM and histogram distribution of UiO-66 nanocrystals synthesized with 1.6 M benzoic acid concentration via hot injection method at 110°C (n = 200 particles).

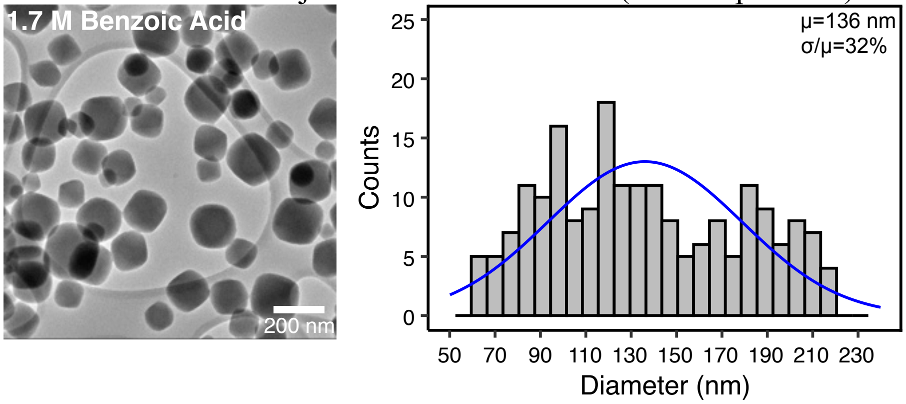

Figure S10. TEM and histogram distribution of UiO-66 nanocrystals synthesized with 1.7 M benzoic acid concentration via hot injection method at 110°C (n = 200 particles).

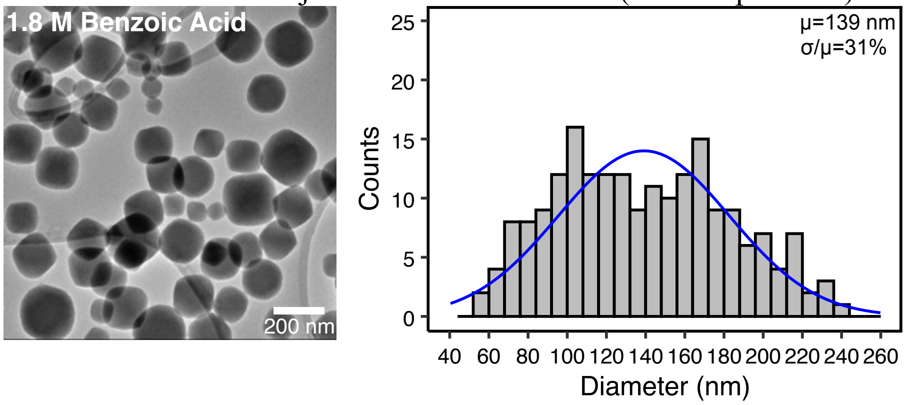

Figure S11. TEM and histogram distribution of UiO-66 nanocrystals synthesized with 1.8 M benzoic acid concentration via hot injection method at 110°C (n = 200 particles).

Evaluation Only. Created with Aspose.Pdf. Copyright 2002-2014 Aspose Pty Ltd.

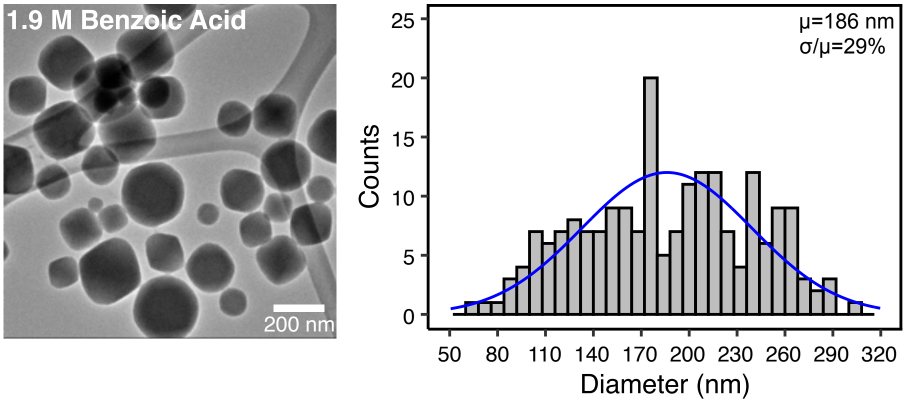

Figure S12. TEM and histogram distribution of UiO-66 nanocrystals synthesized with 1.9 M benzoic acid concentration via hot injection method at 110°C (= 200 particles).

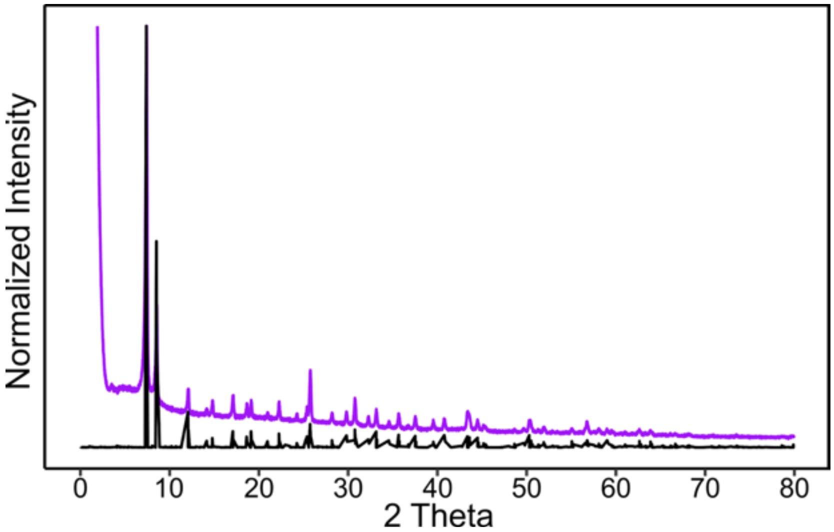

Figure S13. PXRD of UiO-66 nanocrystals synthesized with 1.5 M benzoic acid concentration via hot injection method at 110°C. The PXRD of the nanocrystals after isolation is plotted in purple and simulated UiO-66 diffraction data in black. The diffraction pattern is consistent with that of UiO-66.

Evaluation Only. Created with Aspose.Pdf. Copyright 2002-2014 Aspose Pty Ltd.

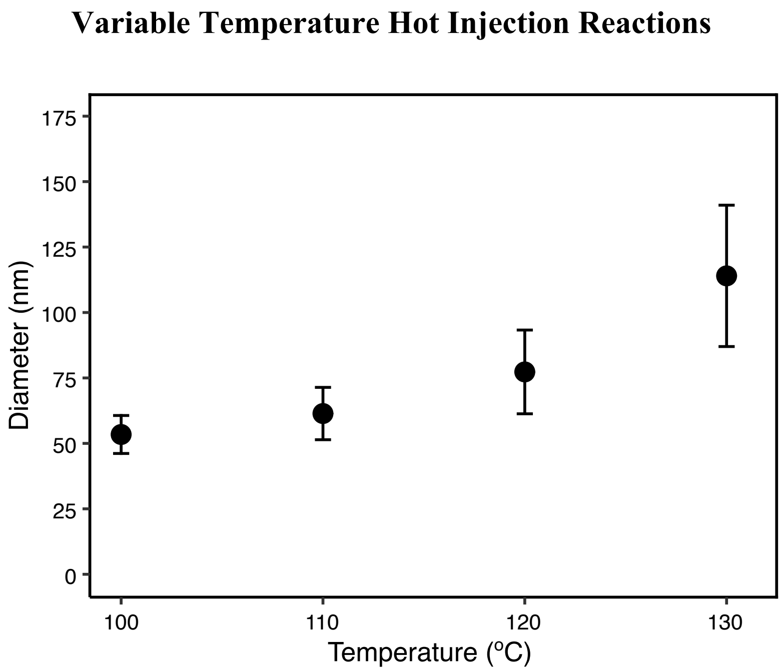

Figure S14. Change in nanocrystal diameter synthesized via the hot injection method with respect to different temperatures at constant 1.4 M benzoic acid. Error bars represent $\mu \pm \sigma$, where μ is the mean diameter and σ is the standard deviation.

Evaluation Only. Created with Aspose.Pdf. Copyright 2002-2014 Aspose Pty Ltd.

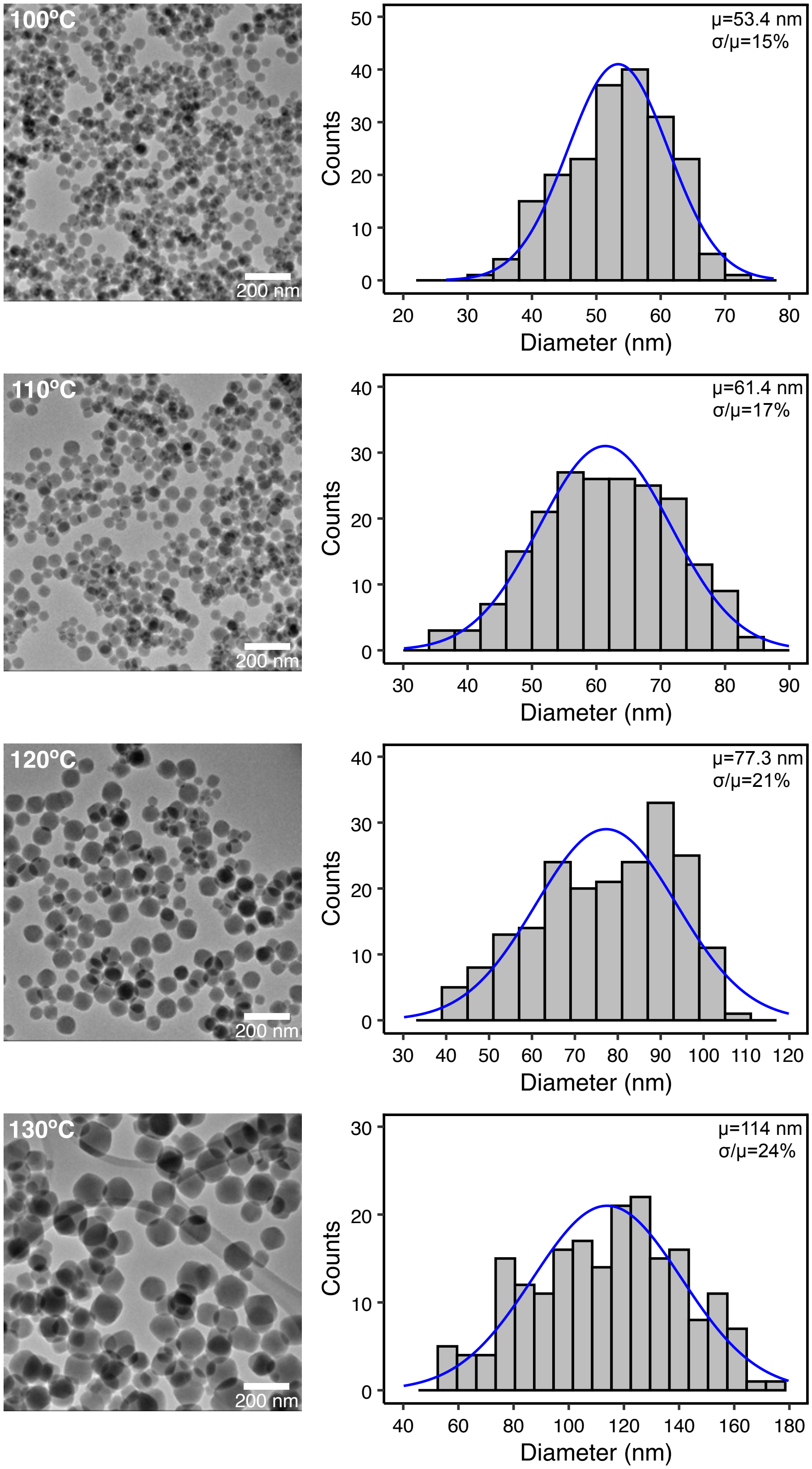

Figure S15. TEM and Histogram UiO-66 nanocrystals synthesized with 1.4 M benzoic acid concentration via hot injection method at various temperatures (n = 200 particles).

Evaluation Only. Created with Aspose.Pdf. Copyright 2002-2014 Aspose Pty Ltd.

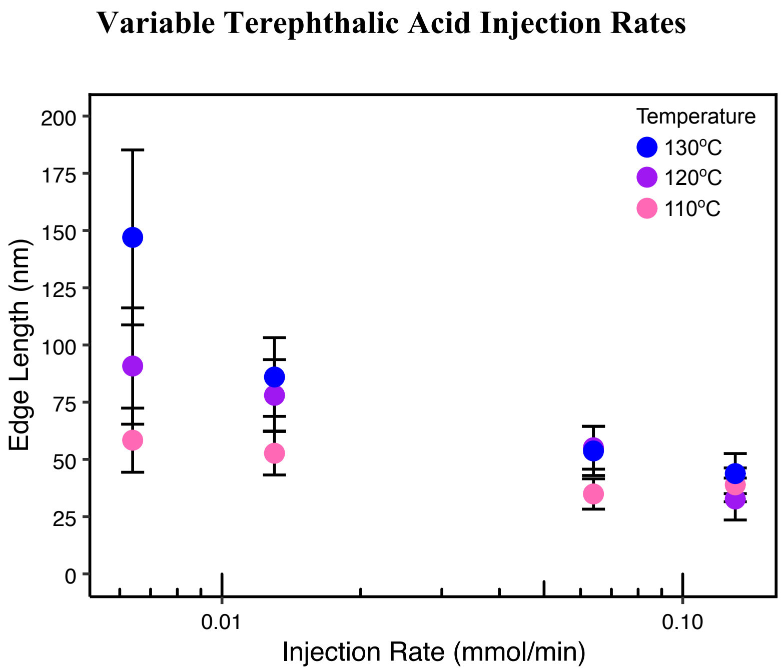

Figure S16. Change in nanocrystal diameter synthesized via the slow injection method with respect to different temperatures at constant 1.4 M benzoic acid. Error bars represent $\mu \pm \sigma$, where μ is the mean diameter and σ is the standard deviation. The edge length is measured rather than the diameter to account for the change in shape from more spherical to octahedral.

Evaluation Only. Created with Aspose.Pdf. Copyright 2002-2014 Aspose Pty Ltd.

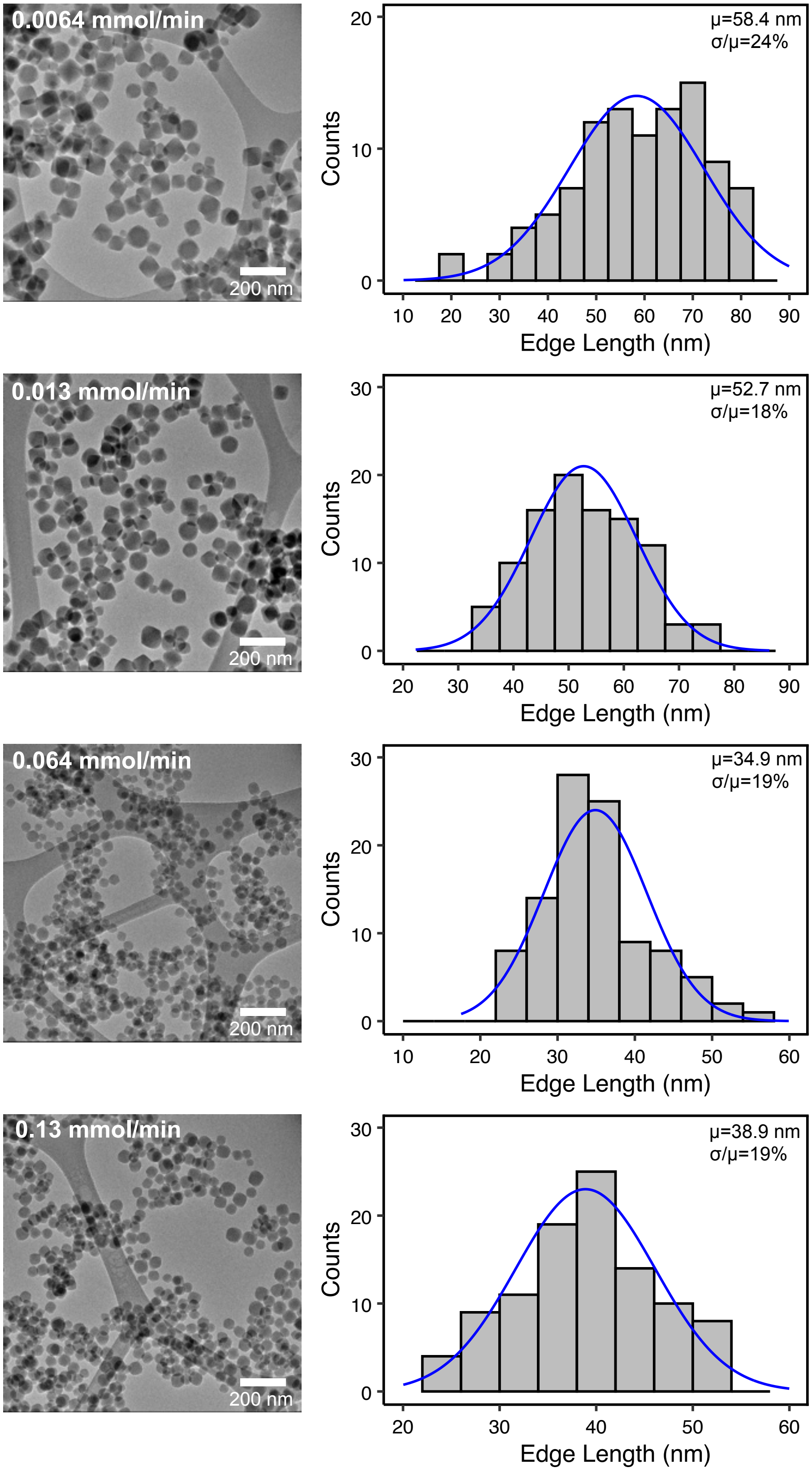

Figure S17. TEM and histogram distribution of UiO-66 nanocrystals synthesized via increasing terephthalic acid injection rate at 110 °C and 1.4 M benzoic acid (n = 100 particles).

Evaluation Only. Created with Aspose.Pdf. Copyright 2002-2014 Aspose Pty Ltd.

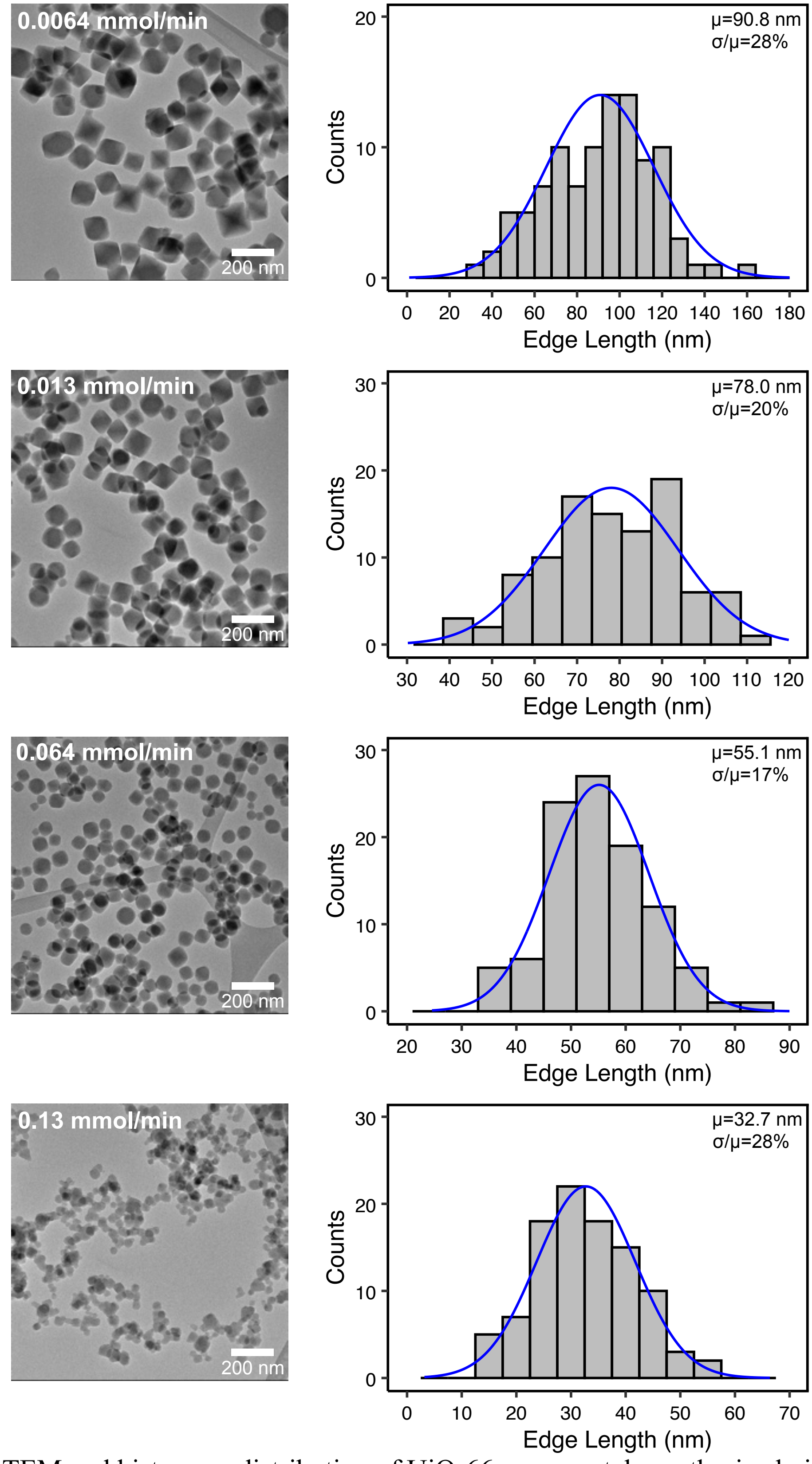

Figure S18. TEM and histogram distribution of UiO-66 nanocrystals synthesized via increasing terephthalic acid injection rate at 120 °C and 1.4 M benzoic acid (n = 100 particles).

Evaluation Only. Created with Aspose.Pdf. Copyright 2002-2014 Aspose Pty Ltd.

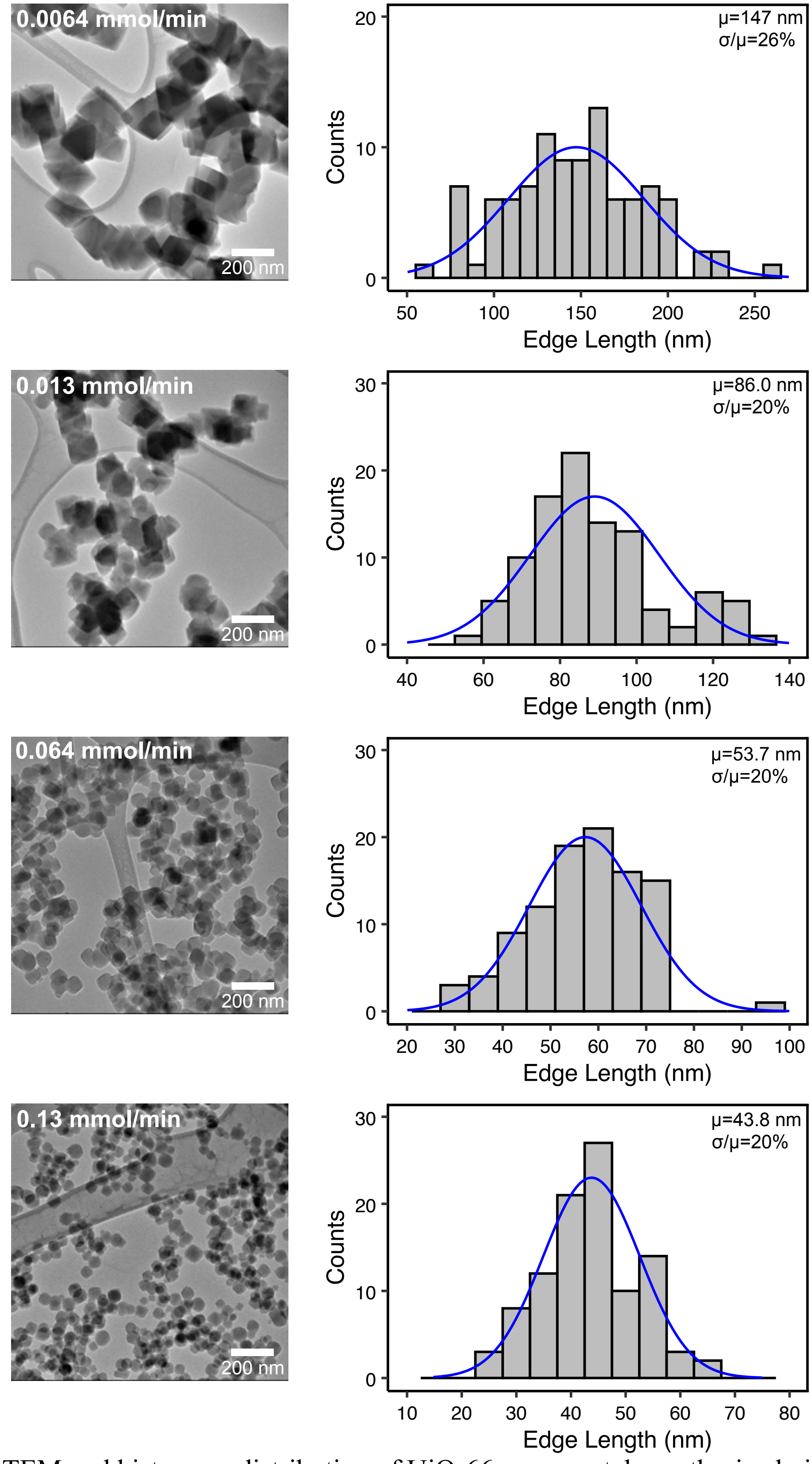

Figure S19. TEM and histogram distribution of UiO-66 nanocrystals synthesized via increasing terephthalic acid injection rate at 130 °C and 1.4 M benzoic acid (n=100 particles).

Evaluation Only. Created with Aspose.Pdf. Copyright 2002-2014 Aspose Pty Ltd.

SECTION S3: YIELD DETERMINATION

The amount of zirconium and the yield of nanocrystals was determined using TGA.

TGA Analysis. A sample of synthesized UiO-66 nanocrystals was loaded onto a clean platinum pan. Under an O_2 flow (50 mL/min), the temperature was raised to 800 °C at a ramp rate of 10 °C /min. Once 800 °C was reached, the temperature was held constant for 10 minutes. The initial and final mass was recorded. The remaining mass is attributed to ZrO_2 and was used to calculate the moles of zirconium according to the method below.

Scheme S1. Decomposition of UiO-66 under O_2 in the TGA instrument.

$$Zr_6O_4(OH)_4(O_4C_8H_4)_6 + 45\ O_2 \xrightarrow{\triangle} 6\ Zr_2O + 48CO_2 + 14H_2O$$

The following sets of equations can be utilized to calculate a percent yield of the MOF via TGA determination of ZrO_2.

$$weight\%_{TGA} * m_{TGA\ crude\ MOF} = m_{TGA\ ZrO_2}$$

$$m_{TGA\ ZrO_2} * \frac{1\ mol\ ZrO_2}{M_{ZrO_2}} * \frac{1\ mol\ [Zr_6O_4(OH)_4]^{12+}}{6\ mol\ ZrO_2} * \frac{M_{[Zr_6O_4(OH)_4]^{12+}}}{1\ mol\ [Zr_6O_4(OH)_4]^{12+}} = m_{TGA\ [Zr_6O_4(OH)_4]^{12+}}$$

$$\frac{m_{TGA\ [Zr_6O_4(OH)_4]^{12+}}}{m_{TGA\ crude\ MOF}} = \%m_{[Zr_6O_4(OH)_4]^{12+}}$$

$$\%m_{[Zr_6O_4(OH)_4]^{12+}} * m_{crude\ MOF} = m_{[Zr_6O_4(OH)_4]^{12+}}$$

$$m_{[Zr_6O_4(OH)_4]^{12+}} * \frac{1\ mol\ [Zr_6O_4(OH)_4]^{12+}}{M_{[Zr_6O_4(OH)_4]^{12+}}} * \frac{1\ mol\ Zr_6O_4(OH)_4(C_8O_4H_4)_6}{1\ mol\ [Zr_6O_4(OH)_4]^{12+}} * \frac{M_{Zr_6O_4(OH)_4(C_8O_4H_4)_6}}{1\ mol\ Zr_6O_4(OH)_4(C_8O_4H_4)_6} = m_{\mathrm{exp}\ Zr_6O_4(OH)_4(C_8O_4H_4)_6}$$

$$\frac{m_{\mathrm{exp}\ Zr_6O_4(OH)_4(C_8O_4H_4)_6}}{m_{\mathrm{actual}\ Zr_6O_4(OH)_4(C_8O_4H_4)_6}} = \%\ yield$$

Evaluation Only. Created with Aspose.Pdf. Copyright 2002-2014 Aspose Pty Ltd.

Table S3. Percent yield calculations for hot injection reactions.

Reaction Temperature (°C)	Modulator Concentration (M)	$\% \, m_{[Zr_6O_4(OH)_4]^{12+}}$	Calculated Percent Yield
110	1.0	23.2%	52%
110	1.1	27.9%	80%
110	1.2	27.5%	22%
110	1.2	31.1%	71%
110	1.4	29.7%	80%
110	1.5	29.7%	73%
110	1.6	31.2%	79%
110	1.7	28.6%	52%
110	1.8	29.2%	44%
100	1.4	29.2%	52%
120	1.4	32.4%	81%
130	1.4	30.0%	56%

Table S4. Percent yield calculations for slow injection reactions.

Reaction Temperature (°C)	Injection Rate (mmol/min)	$\% \, m_{[Zr_6O_4(OH)_4]^{12+}}$	Calculated Percent Yield
110	0.0064	32.5%	80%
110	0.013	29.7%	82%
110	0.064	30.9%	55%
110	0.13	31.4%	60%
120	0.0064	28.4%	80%
120	0.013	27.2%	65%
120	0.064	32.9%	61%
120	0.13	32.5%	71%
130	0.0064	29.7%	63%
130	0.013	30.6%	65%
130	0.064	32.5%	67%
130	0.13	32.0%	66%

Evaluation Only. Created with Aspose.Pdf. Copyright 2002-2014 Aspose Pty Ltd.

SECTION S4. NANOCRYSTAL SOLUBILITY MEASUREMENTS

^{13}C-Labeled UiO-66 Nanocrystals. ^{13}C-labeled UiO-66 nanocrystals were synthesized using the hot injection method described in the experimental section and terephthalic acid-2,2'-$^{13}C_2$.

^{13}C NMR Dissolution Measurements. To a 20 mL vial, ^{13}C-labeled UiO-66 (0.0217 g, 0.152 mmol), benzoic acid (0.1607 g, 1.32 mmol), and 5.5 mL of anhydrous DMF were added. Assuming 1675.9 g/mol molar mass of the ^{13}C-labeled UiO-66 material, the concentration of Zr in the solution was equal to 0.014 M, consistent with the UiO-66 nanocrystal synthetic methods described in this work. The reaction was stirred and heated at 110 °C on a metal heating plate for 5 minutes before being removed from the heat and briefly sonicated. This resulted in a cloudy solution of UiO-66 nanocrystals.

In separate 4 mL vials, utilizing a Hamilton syringe, 500 µL of the MOF solution were added to increasing amount of benzoic acid, such that when the 500 µL MOF solution is added, the total benzoic acid concentration would be equal to 1.0 M, 1.3 M, 1.5 M, 1.7 M. 1.9 M, 2.2 M, 2.5 M, 2.7 M, 3.0 M, 3.5 M, or 6.0 M in DMF. The solutions were heated and stirred at 110 °C on a metal heating plate for 5 minutes before being removed from the heat and transferred to an NMR tube. The procedure was repeated for nanocrystals with diameters 26 nm, 36 nm, 52.9 nm, and 108 nm. The solution decreases in cloudiness and results in a clear, homogeneous solutions 3.0 M and above for all nanocrystal sizes.

{^{1}H} ^{13}C NMR spectra were collected using a Brucker 500 MHz spectrometer. The signal from d_8-Toluene of a known concentration was used as an internal standard and used to normalize the integral of the reference peak at δ = 137.8 ppm. Integration values of the terephthalic acid and the internal standard were used to calculate the relative concentration of free terephthalic acid. The T_1 time of the terephthalic acid was measured to using the inversion recovery method and found to be 6 seconds. The relaxation delay was set to $T_1 * 5 = 30$ seconds and the decoupler left on during the relaxation delay. As an additional check, we performed several measurements with a range of relaxation times varying from 30 to 120 seconds and found no/insignificant difference in the integral recorded.

Evaluation Only. Created with Aspose.Pdf. Copyright 2002-2014 Aspose Pty Ltd.

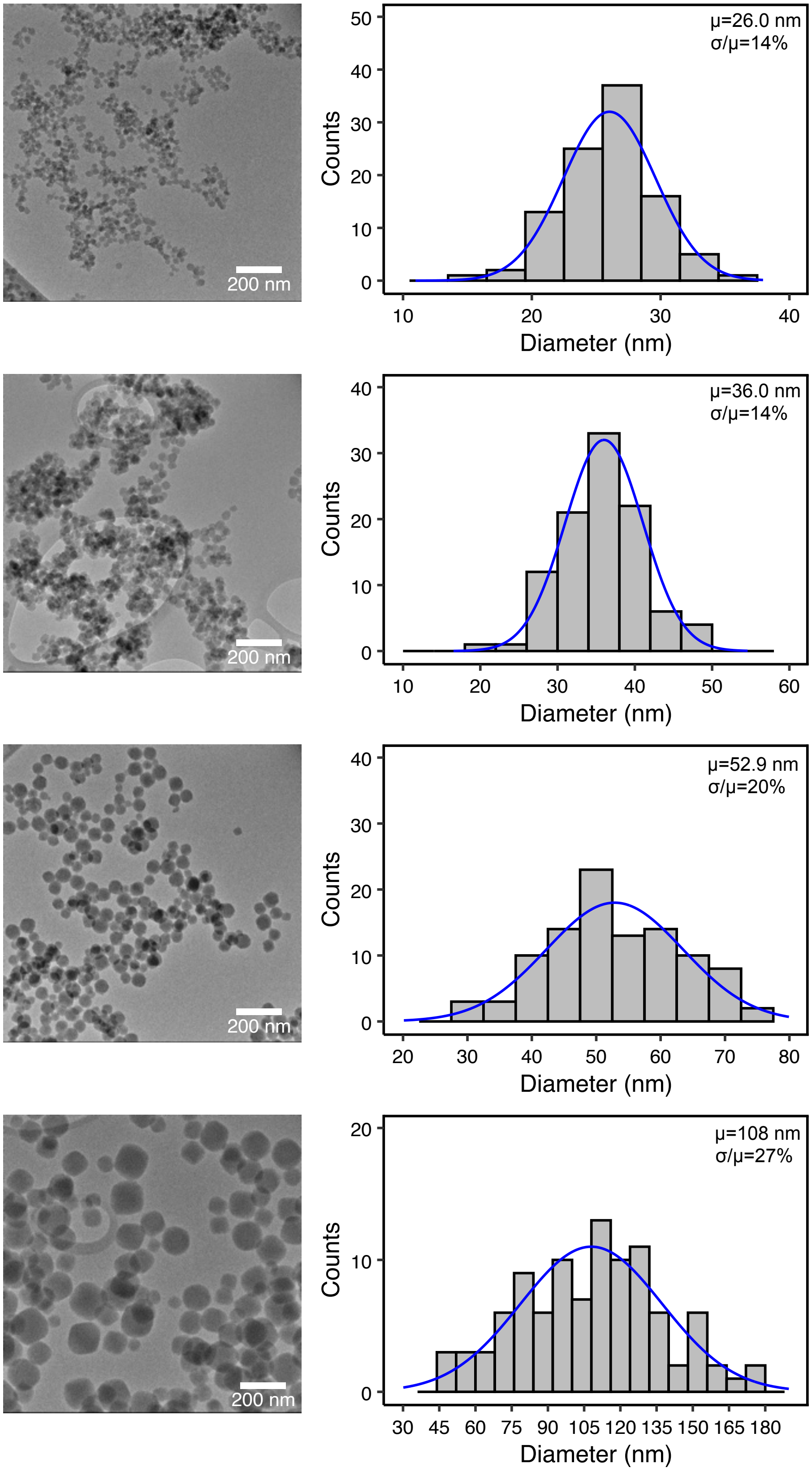

Figure S20. TEM and Histogram UiO-66 nanocrystals synthesized with terephthalic acid-2,2'-$^{13}C_2$ at various sizes (n =100 particles).

Evaluation Only. Created with Aspose.Pdf. Copyright 2002-2014 Aspose Pty Ltd.

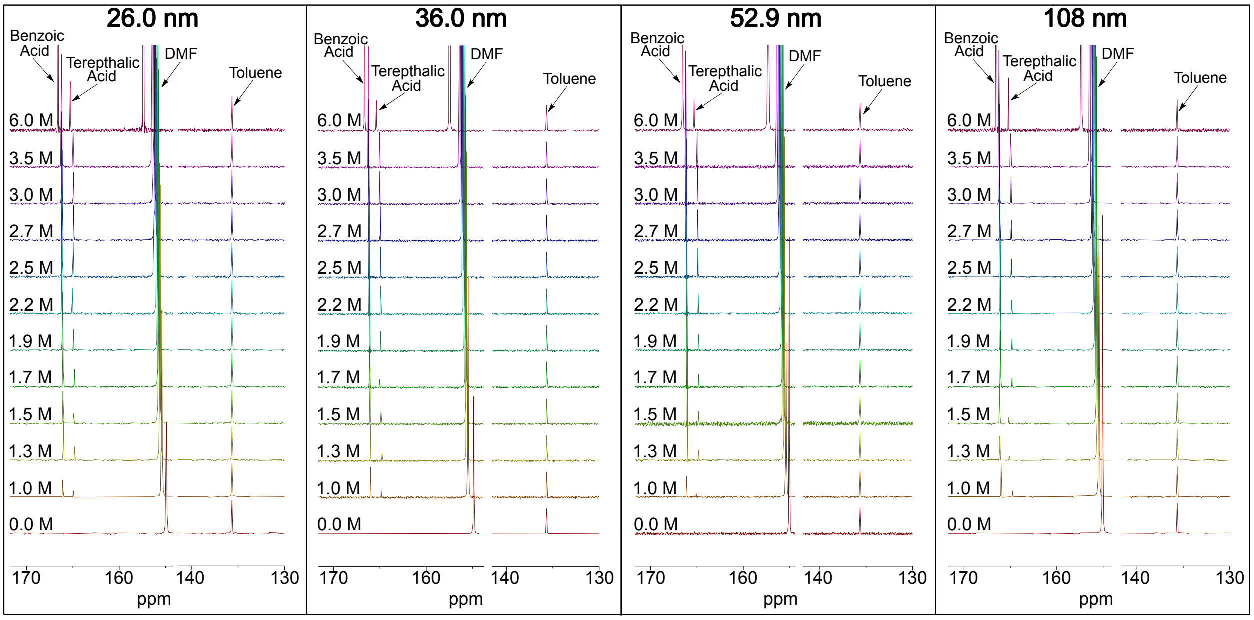

Figure S21. Annotated ^{13}C NMR spectra of MOF nanocrystals at various sizes treated with benzoic acid. Toluene-d_8 was utilized as an internal standard.

The change in the terephthalic acid concentration with increasing benzoic acid was fit to a four-parameter logistic regression for each nanocrystal size,

$$y = d + \frac{(a - d)}{1 + (\frac{x}{EC50})^h}$$

where *d* is the maximum asymptote, *a* is the minimum asymptote, *EC50* is the inflection point, and *h* is the slope factor. The minimum asymptote values were fixed to the lowest terephthalic acid concentration recorded in the dissolution measurements for each nanocrystal size. An excess of benzoic acid of 6 M was utilized to dissolve the UiO-66 nanocrystals, and the measured terephthalic concentration via ^{13}C NMR was used as the fitting parameter for the maximum asymptote.

A clear trend is observed in the minimum asymptote variable, as can be seen by the change in the intercept at [benzoic acid] = 0 in **Figure 2B**. An increase, followed by a plateau of terephthalic acid concentration, is observed that is size dependent. Assuming prior to the first plateau is the release of surface ligands, we can estimate the percentage of surface terephthalates on each nanocrystal size as 28.1% for 26 nm particles, 21.5% for 36 nm particles, 17.0% for 52.9 nm particles and 10.7% for 108 nm particles. Compared to the modeled, calculated values summarized in **Table S5**, the experimental versus theoretical values are within 10% difference, within the NMR integration error. Therefore, it is a fair argument to suggest that prior to the first plateau, surface terephthalates are participating in an exchange with benzoic acid, contributing to the initial increase in free terephthalic acid ^{13}C signal.

Evaluation Only. Created with Aspose.Pdf. Copyright 2002-2014 Aspose Pty Ltd.

Table S5. Surface terephthalic acid percentage calculation.

Diameter (nm)	Number of Unit Cells in Cubic MOF	Total # Linkers	# Labile Carboxylates on Surface Node Corner	# Labile Carboxylates on Surface Node Edge	# Labile Carboxylates on Surface Node Face	Total # Labile Carboxylates	% Labile Carboxylates (modeled)	% Surface Terephthalates (Experimental)
26	9 x 9	25674	72	672	4350	5094	19.8	28.1
36	13 x 13	69522	72	1008	9390	10470	15.0	21.5
52.9	19 x 19	200094	72	1512	20550	22134	11.1	17
108	37 x 37	1348674	72	3024	79950	83046	6.1	10.7

To differentiate simple surface ligand exchange versus actual dissolution of the interior MOF framework, the lower asymptote is utilized as the baseline, and the surface terephthalate concentration is subtracted from further analysis in both K_{sp} and surface tension calculations.

Table S6. Summary of the fitted *EC50* values, *h*, and R^2 for each fit.

Nanocrystal Diameter (nm)	Benzoic Acid (M) at *EC50*	Terephthalic Acid (mM) at EC50*	Terephthalic Acid (mM) at *EC50*$^+$	Slope factor (*h*)	R^2
26.0	2.022	7.06	3.960	7.704	0.974
36.0	2.106	6.445	4.165	6.533	0.980
52.9	2.473	6.445	4.575	8.024	0.987
108	3.078	7.485	6.035	5.702	0.996

* Terephthalic Acid (mM) at EC50 includes surface terephthalate concentration
$^+$Terephthalic Acid (mM) at EC50 does not include surface terephthalate concentrations. These EC50 values are used in further calculations.

K_{sp} values for each nanocrystal size were determined at the EC50 induction point. The RICE table below is the equilibrium equation of dissolution of the UiO-66 nanocrystal. $Zr_6O_4(OH)_4(T)_6$, where *T* is a terephthalate ligand, reacts with twelve equivalents of *BA*, benzoic acid. A ligand exchange of *T* to *B*, benzoate ligand, is achieved, resulting in the dissolution of the nanocrystal to a hexa-zirconium cluster and release of *TA*, terephthalic acid, into solution. *X* is the initial benzoic acid concentration.

Table S7. RICE equilibrium table for the acidolysis of UiO-66 nanocrystals.

**Reaction**	$Zr_6O_4(OH)_4(T)_6$	+	$12\,BA$	$\longrightarrow$	$[Zr_6O_4(OH)_4(B)_{12}]$	+	$6\,TA$
**Initial**	*solid*		X		0		0
**Change**	*solid*		$-12S$		$+S$		$+6S$
**Equilibrium**	*solid*		$X-12S$		S		$[TA]$

Using the dissolution equation described above, the following equation can be written to calculate for the K_{sp} of the UiO-66 nanocrystal.

Evaluation Only. Created with Aspose.Pdf. Copyright 2002-2014 Aspose Pty Ltd.

$$K_{eq} = \frac{[Zr_6O_4(OH)_4(B)_{12}][TA]^6}{[Zr_6O_4(OH)_4(T)_6][BA]^{12}}$$

From our K_{sp} study, we aimed to investigate the surface energy of the nanocrystal at various sizes. The Ostwald-Freundlich equation,

$$\frac{S}{S_o} = e^{\frac{2*\gamma*V_m}{R*T*r}}$$

where S is the solubility of the specific nanocrystal in molkg^{-1}, S_o is the bulk solubility in molkg^{-1}, γ is the surface free energy or surface tension in Jm^{-2}, V_m is the molar volume in m^{3}mol^{-1}, R is the Jmol^{-1}K^{-1}, T is the temperature in K, and r is the radius in m, relates the solubility to the surface tension of the nanocrystals.[4,5] This equation can be rearranged to a linear form:

$$ln(S) = \frac{2\gamma V_m}{RT} * \frac{1}{r} + ln\,(S_o)$$

The ln(S) versus r^{-1} can be plotted for each nanocrystal size and added [benzoic acid] (M). By fitting a linear regression through each benzoic acid concentration, the slope of the line can be used to calculate the surface tension of the nanocrystals (**Figure S27**). The calculated surface tension values at each benzoic acid concentration are shown in **Figure 3C**. Larger uncertainties are observed at lower benzoic acid concentrations due to the extremely low solubility at these data points. As stated above, surface ligands are not included in the calculations. Additionally, by identifying the y-intercept, or the ln(S_o), the bulk solubility of the UiO-66 MOF at each [benzoic acid] can be calculated in molkg^{-1}. Using conversion factors, the solubility in terms of mol/L is calculated, and the concentration of terephthalic acid (mM) at each [benzoic acid] (M) is predicted (**Figure 3D**). The terephthalic acid concentration points are fit to a four-parameter logistic curve, and the midpoint, or EC50 value, is identified (**Table S7**), in which the bulk K_{sp} is calculated according to **Table S6**.

Table S8. Summary of the fitted *EC50* value, *h*, and R^2 for bulk UiO-66.

Nanocrystal Diameter (nm)	Benzoic Acid (M) at *EC50*	Terephthalic Acid (mM) at EC50	Slope factor (*h*)	R^2
Bulk	3.026	5.83	6.977	0.999

Evaluation Only. Created with Aspose.Pdf. Copyright 2002-2014 Aspose Pty Ltd.

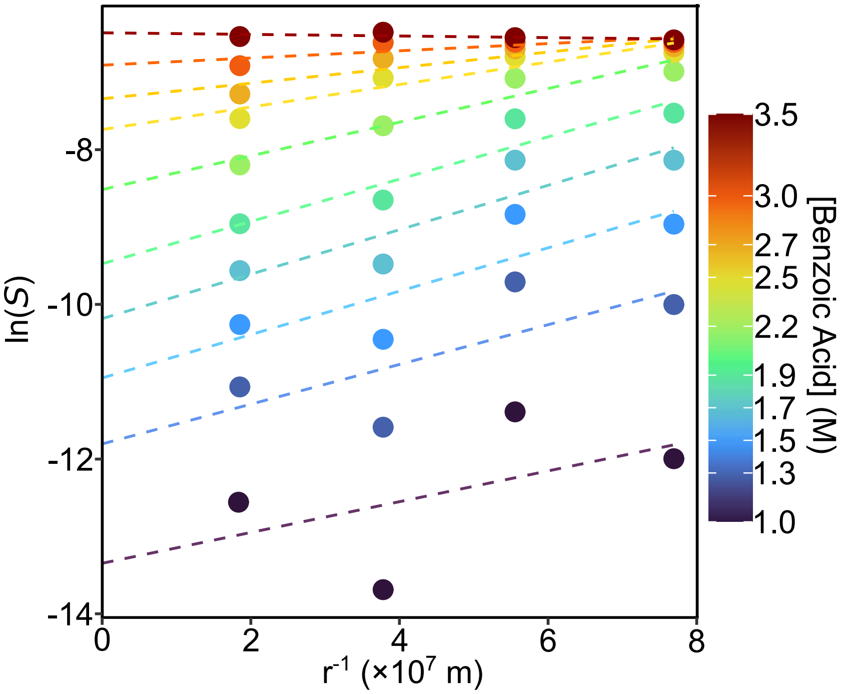

Figure S22. The natural log of the solubility vs r^{-1} at each nanocrystal size and [Benzoic Acid] (M). Dashed lines are a linear regression fit.

However, we note that Ostwald-Freundlich relationship assumes spherical nanocrystals, not accounting for distinct crystal facets, neglects aggregation effects, and relies on a molar volume derived from bulk UiO-66 crystal structure data, all of which contribute uncertainty in the precise surface energy value.[4,5]

Ostwald Ripening During Solubility Measurements. To verify the lack of Ostwald ripening during dissolution experiments, aliquots were taken and the nanocrystals imaged via TEM. No significant size broadening is occurring during dissolution.

Evaluation Only. Created with Aspose.Pdf. Copyright 2002-2014 Aspose Pty Ltd.

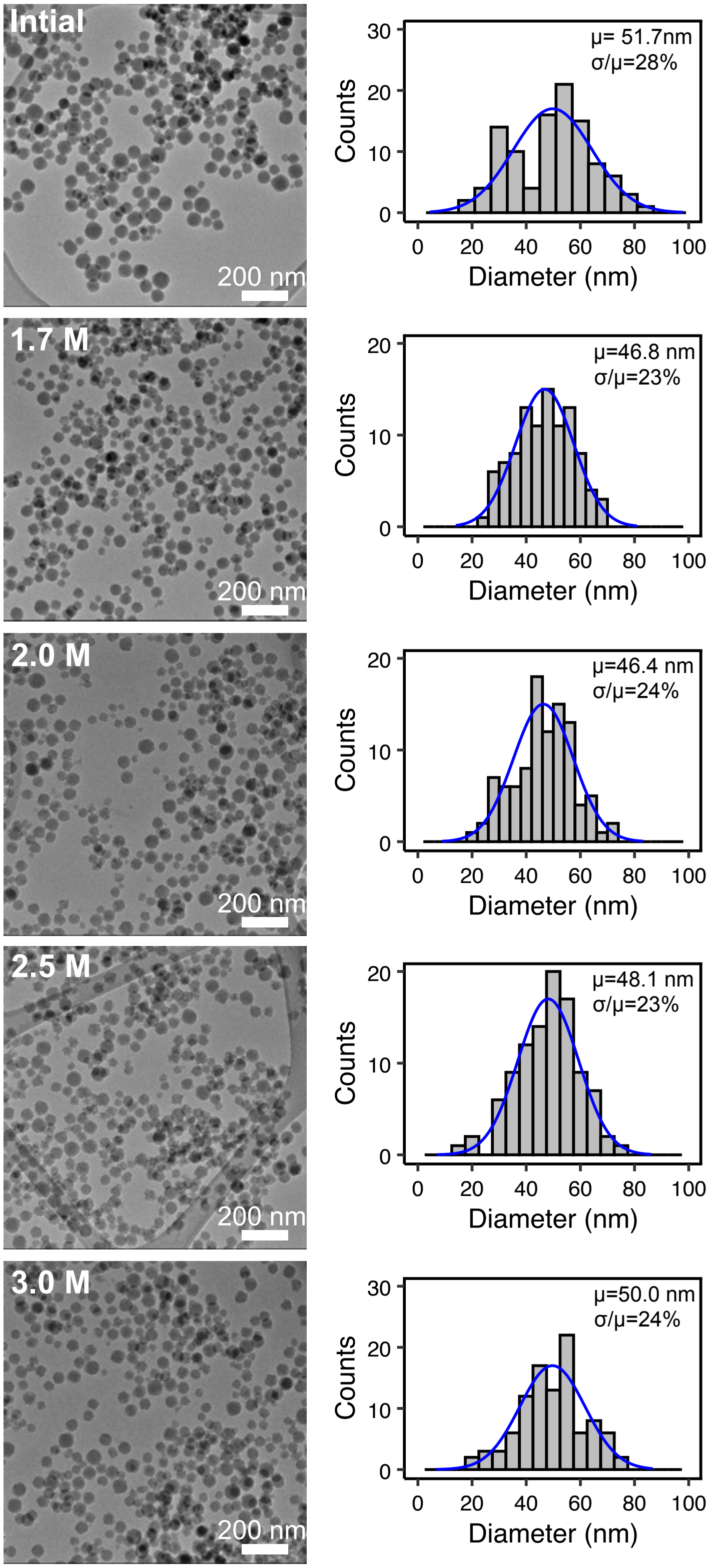

Figure S23. TEM images of dissolution of nanocrystals at multiple benzoic acid concentrations (n=100 particles).

Evaluation Only. Created with Aspose.Pdf. Copyright 2002-2014 Aspose Pty Ltd.

Long Term Ostwald Ripening Study. To explore ripening over timescales that are consistent with conventional syntheses beginning from ZrOCl and much longer than the hot injection syntheses used here, a nanoMOF synthesis conducted at [benzoic] = 1.5 M was allowed to heat on a metal heating plate for 24 hours prior to isolation via the described washing methods (see experimental section 2.2.2 in the supporting information). The particle size remains within 5 nm (μ = 90.6 nm for method described in section 2.2.2 in the SI, and μ = 85.9 nm for 24 hour reaction time) , polydispersity within 2% (μ/σ = 22% for method described in section 2.2.2 in the SI, and μ/σ = 24% for 24 hour reaction time), and isolated yield within 8% (73% for method described in section 2.2.2 in the SI, and 81% for 24 hour reaction time). The isolated yield is calculated via TGA described in section S3 of the supporting information. The lack of significant change in particle size, polydispersity, and yield after 24 hours indicates that Ostwald ripening of UiO-66 in benzoic acid and DMF is negligible.

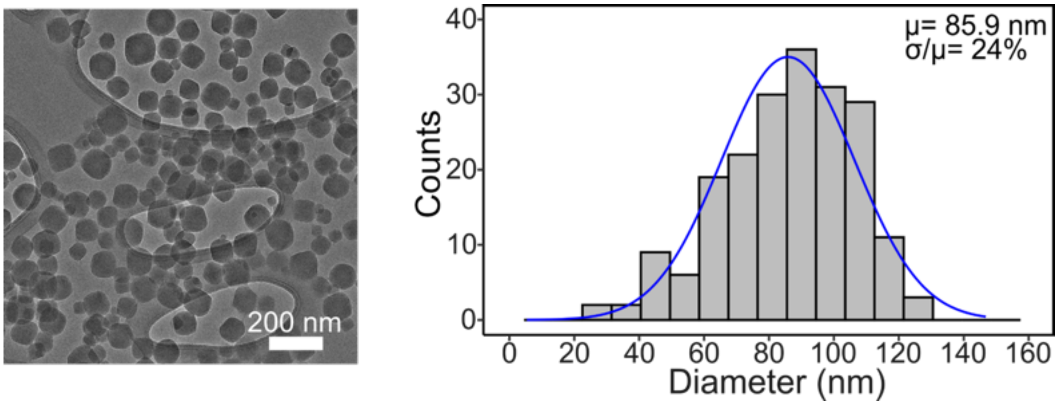

Figure S24. TEM and histogram distribution of UiO-66 nanocrystals synthesized with 1.5 M benzoic acid concentration via hot injection method at 110 °C (n=200 particles) after 24 hours of reaction time. The particle size is equivalent to the 5 min. reaction time, which is consistent with slow Ostwald ripening kinetics.

Additional MOF loading. To verify that the MOF dissolution had reached quasi-equilibrium, the influence of the MOF loading on the K_{sp} was measured. To a 4 mL glass vial, 4.0 mg of ^{13}C-labeled MOF (diameter = 52.9 nm), 1 mL of DMF, and benzoic acid (0.3020 g, 2.47 mmol) were added. The vial is capped, and heated at 110 °C on a metal heating mantle while stirring at 350 rpm. The vial was removed from heat, 400 µL of d_8-toluene was added, and reaction solution was transferred to a J-Young NMR tube. The acquired spectrum is labeled Initial in **Figure S26**. Next, an additional 6.8 mg of ^{13}C-labeled MOF (diameter = 52.9 nm) was added to the NMR tube and the mixture heated in an oil bath at 110 °C for an additional 5 minutes. The vial was removed from heat, and the ^{13}C NMR spectrum acquired.

Figure S25 shows a small increase in the [terephthalic acid] from 4.8 mM in the Initial spectrum to 6.9 mM in Added MOF spectrum. Accounting for surface ligands extracted from the intercept in **Figure 2B** of the main text and described in Section S4 of the Supporting Information, a 3.1 mM increase in [terephthalic acid] would be expected solely from the surface carboxylate exchange. Thus, the observed increase of 2.1 mM is smaller than the surface ligand amount, indicating that no additional MOF dissolves. This verifies that solubility products measured here are not proportional to the surface area of the MOFs, as was suggested by a reviewer.

Evaluation Only. Created with Aspose.Pdf. Copyright 2002-2014 Aspose Pty Ltd.

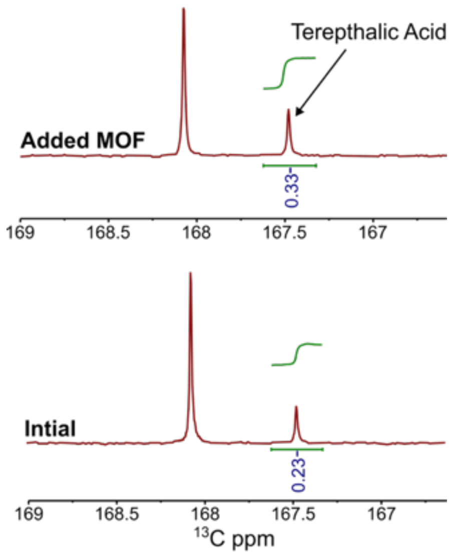

Figure S25. ^{13}C NMR spectra after initial dissolution and additional MOF loading. The increase in the integration is due to the partial liberation of surface terephthalate ligands (See **Figure 2B** and section S4 for added discussion).

Reversibility of Precipitation. To test the reversibility of the MOF dissolution equilibrium, a DMF solution of terephthalic acid is added to solutions of MOFs that had been previously dissolved in benzoic acid. The added terephthalic acid caused the precipitation of UiO-66 as was verified by powder X-ray diffraction. The formation of MOFs from the benzoic acid solution is consistent with a reversible precipitation equilibrium rather than an irreversible decomposition of the MOF. Given the short time scale of these experiments, we conclude that the nodal clusters likely remain intact during dissolution.

To a 20 mL glass vial, 21.0 mg of UiO-66 nanocrystals (90.4 nm in diameter), benzoic acid (1.9233 g, 15.7 mmol) and 5.25 mL of anhydrous DMF were added. The reaction was stirred at 350 rpm and heated at 110 °C on a metal heating plate for 5 minutes. At this [benzoic acid] (3M) more than 50% of the MOF framework dissolves resulting in a translucent dispersion (**Figure S27A**). The dissolution of the particles is apparent from the loss of cloudy precipitate (**Figure S27**).

Terephthalic acid (0.0735 g, 0.442 mmol, 5.8 equiv per Zr) was added to the sample, and the mixture stirred and heated at 110 °C on a metal heating plate for 10 minutes. A white precipitate forms resulting in a cloudy mixture (**Figure 27B**). The precipitate is isolated via the washing procedures in the experimental section 2.2.2 of the supporting information. 0.0164 grams or 78% of the original MOF mass was recovered and analyzed by powder x-ray diffraction, verifying the newly formed precipitate is UiO-66. A theoretical yield of 99% of isolated MOF is expected after addition of terephthalic acid.

Evaluation Only. Created with Aspose.Pdf. Copyright 2002-2014 Aspose Pty Ltd.

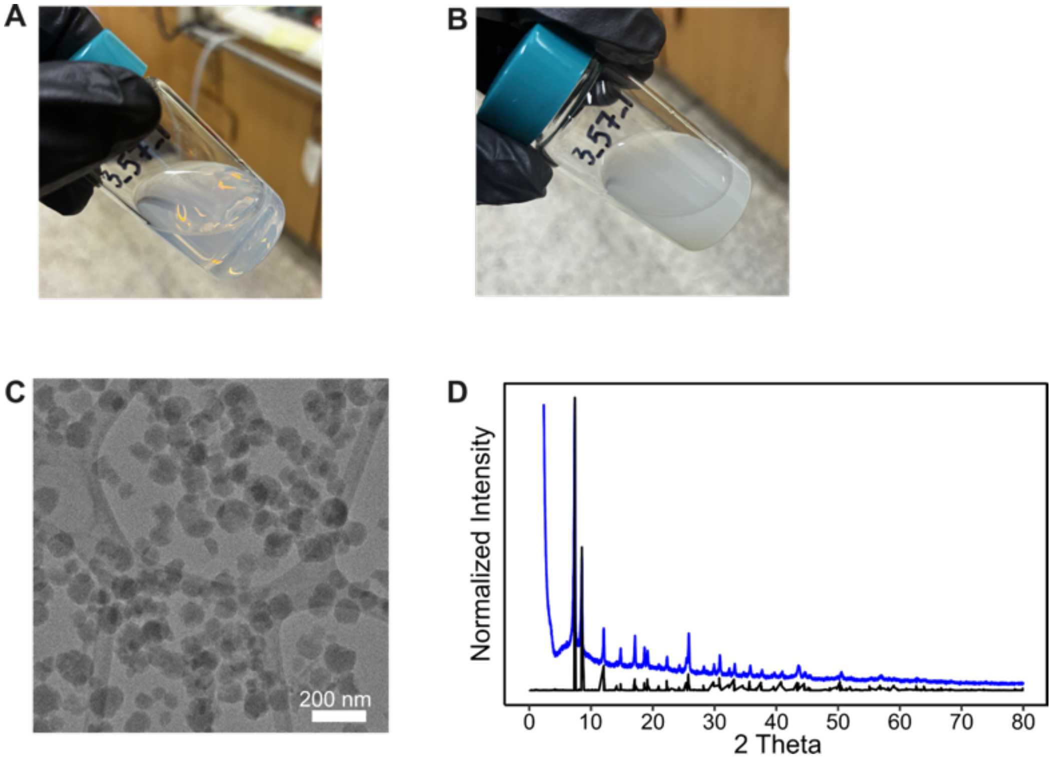

Figure S26. (A) The reaction solution after dissolution of the MOF nanocrystals at 3M benzoic acid. (B) The precipitation of the MOF nanocrystals at 3M benzoic acid with an increase in terephthalic acid. (C) TEM characterization of the nanocrystals after isolation. (D) PXRD of the nanocrystals after isolation is plotted in blue and simulated UiO-66 diffraction data in black. The diffraction pattern is consistent with that of UiO-66.

Evaluation Only. Created with Aspose.Pdf. Copyright 2002-2014 Aspose Pty Ltd.

SECTION S5: MODELING

Modeling was completed utilizing the cubic unit cell of UiO-66 available via the Cambridge Crystallographic Data Centre (CCDC).[6] Depending on the shape of the nanocrystal, either a cubic shape or octahedral model was utilized. An octahedral nanocrystal was modeled by truncating the cubic structure at the vertices parallel to the [111] facets, as detailed in the literature,[3] in the publicly available software VESTA.[7]

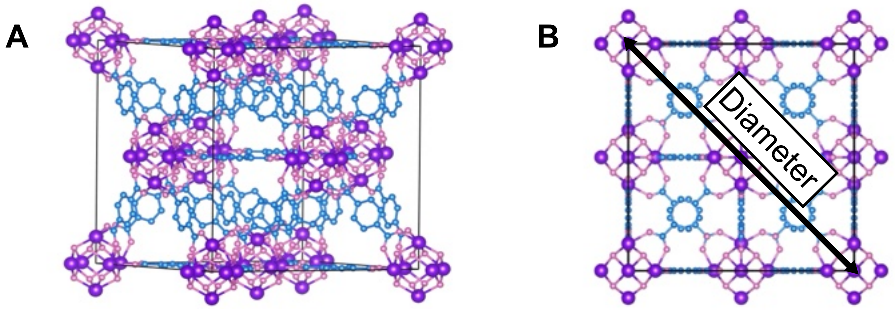

Figure S27. (A) UiO-66 cubic nanocrystal model. (B) Diameter measurement of cubic nanocrystals.

Evaluation Only. Created with Aspose.Pdf. Copyright 2002-2014 Aspose Pty Ltd.

Table S9. Modeling calculations of UiO-66 nanocrystals with a cubic shape.

Number of Unit Cells in Cubic MOF	Edge Length of Cubic MOF (nm)	Diameter Length of Cubic MOF (nm)	# Surface Nodes (cubic MOF)	# Interior Nodes (cubic MOF)	# Total Nodes (cubic MOF)
1x1x1	2.075	2.927	14	0	14
2x2x2	4.149	5.855	50	13	63
3x3x3	6.224	8.782	110	62	172
4x4x4	8.299	11.71	194	171	365
5x5x5	10.37	14.64	326	400	726
6x6x6	12.45	17.56	434	665	1099
7x7x7	14.52	20.49	590	1098	1688
8x8x8	16.60	23.42	770	1687	2457
9x9x9	18.67	26.35	974	2456	3430
10x10x10	20.75	29.27	1202	3429	4631
11x11x11	22.82	32.20	1454	4630	6084
12x12x12	24.90	35.13	1730	6083	7813
13x13x13	26.97	38.06	2030	7812	9842
14x14x14	29.06	40.98	2354	9841	12195
15x15x15	31.12	43.91	2702	12194	14896
16x16x16	33.19	46.84	3074	14895	17969
17x17x17	35.27	49.77	3470	17968	21438
18x18x18	37.34	52.69	3890	21437	25327
19x19x19	39.42	55.62	4334	25326	29660
20x20x20	41.49	58.59	4802	29659	34461
21x21x21	43.57	61.48	5294	34460	39754
22x22x22	45.64	64.40	5810	39753	45563
23x23x23	47.72	67.33	6350	45562	51912
24x24x24	49.79	70.26	6914	51911	58825
25x25x25	51.87	73.19	7502	58824	66326
26x26x26	53.94	76.11	8114	66325	74439
27x27x27	56.02	79.04	8750	74438	83188
28x28x28	58.09	81.97	9410	83187	92597
29x29x29	60.16	84.90	10094	92596	102690
30x30x30	62.24	87.82	10802	102689	113491
nxnxn	$n * 2.075$	$n*2.927$	$12n^2+2$	$4n^3+2n^2+n-1$	# Surface + # Interior Nodes

Evaluation Only. Created with Aspose.Pdf. Copyright 2002-2014 Aspose Pty Ltd.

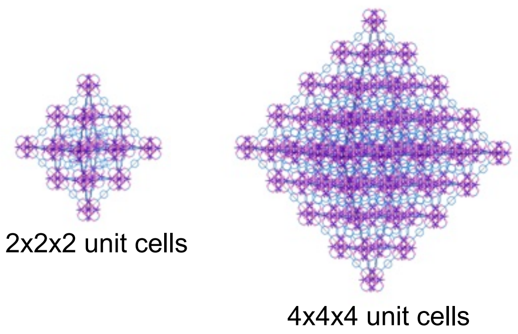

Figure S28. Examples of UiO-66 octahedral nanocrystal models.

Evaluation Only. Created with Aspose.Pdf. Copyright 2002-2014 Aspose Pty Ltd.

Table S10. Modeling calculations for UiO-66 nanocrystals of octahedral shape

Number of Unit Cells in Cubic MOF	Edge Length of Cubic MOF (nm)	Edge Length of octahedral MOF (nm)[3]	# Surface Nodes (octahedral MOF)	# Interior Nodes (octahedral MOF)	# Total Nodes (octahedral MOF)
1x1x1	2.075	1.467	6	0	6
2x2x2	4.149	2.934	18	1	19
3x3x3	6.224	4.401	38	6	44
4x4x4	8.299	5.868	66	19	85
5x5x5	10.37	7.335	102	44	146
6x6x6	12.45	8.802	146	85	231
7x7x7	14.52	10.27	198	146	344
8x8x8	16.60	11.74	258	231	489
9x9x9	18.67	13.20	326	344	670
10x10x10	20.75	14.67	402	489	891
11x11x11	22.82	16.14	486	670	1156
12x12x12	24.90	17.60	578	891	1469
13x13x13	26.97	19.07	678	1156	1834
14x14x14	29.06	20.54	786	1469	2255
15x15x15	31.12	22.00	902	1834	2736
16x16x16	33.19	23.47	1026	2255	3281
17x17x17	35.27	24.94	1158	2736	3894
18x18x18	37.34	26.41	1298	3281	4579
19x19x19	39.42	27.87	1446	3894	5340
20x20x20	41.49	29.34	1602	4579	6181
21x21x21	43.57	30.81	1766	5340	7106
22x22x22	45.64	32.27	1938	6181	8119
23x23x23	47.72	33.74	2118	7106	9224
24x24x24	49.79	35.21	2306	8119	10425
25x25x25	51.87	36.68	2502	9224	11726
26x26x26	53.94	38.14	2706	10425	13131
27x27x27	56.02	39.61	2918	11726	14644
28x28x28	58.09	41.08	3138	13131	16269
29x29x29	60.16	42.54	3366	14644	18010
30x30x30	62.24	44.01	3602	16269	19871
nxnxn	$n * 2.075$	$\frac{2.075 * n}{\sqrt{2}}$	$4n^2 + 2$	$(n-1)^2 + (2 * (n-2)^2) + (2(n-3)^2) + \cdots + (0)^2$	# Surface + # Interior Nodes

Evaluation Only. Created with Aspose.Pdf. Copyright 2002-2014 Aspose Pty Ltd.

References

(1) Baranov, D.; Lynch, M. J.; Curtis, A. C.; Carollo, A. R.; Douglass, C. R.; Mateo-Tejada, A. M.; Jonas, D. M. Purification of Oleylamine for Materials Synthesis and Spectroscopic Diagnostics for Trans Isomers. *Chem. Mater.* **2019**, *31* (4), 1223–1230.

(2) Jerozal, R. T.; Pitt, T. A.; MacMillan, S. N.; Milner, P. J. High-Concentration Self-Assembly of Zirconium- and Hafnium-Based Metal–Organic Materials. *J. Am. Chem. Soc.* **2023**, *145* (24), 13273–13283.

(3) Park, S. V.; Bhai, L.; Lee, A.; Park, A.-H. A.; Marbella, L. E.; Owen, J. S. Steric Stabilization of Colloidal UiO-66 Nanocrystals with Oleylammonium Octadecylphosphonate. *Chem. Sci.* **2025**, *16* (2), 933–938.

(4) Mudunkotuwa, I. A.; Rupasinghe, T.; Wu, C.-M.; Grassian, V. H. Dissolution of ZnO Nanocrystals at Circumneutral pH: A Study of Size Effects in the Presence and Absence of Citric Acid. *Langmuir* **2012**, *28* (1), 396–403.

(5) Liu, J.; Aruguete, D. M.; Murayama, M.; Hochella, M. F., Jr. Influence of Size and Aggregation on the Reactivity of an Environmentally and Industrially Relevant Nanomaterial (PbS). *Environ. Sci. Technol.* **2009**, *43* (21), 8178–8183.

(6) Cavka, J. H.; Jakobsen, S.; Olsbye, U.; Guillou, N.; Lamberti, C.; Bordiga, S.; Lillerud, K. P. A New Zirconium Inorganic Building Brick Forming Metal Organic Frameworks with Exceptional Stability. *J. Am. Chem. Soc.* **2008**, *130* (42), 13850–13851.

(7) Momma, K.; Izumi, F. *J. Appl. Crystallogr.* **2011**, *44*, 1272–1276.
